# Burst control: Synaptic conditions for burst generation in cortical layer 5 pyramidal neurons

**DOI:** 10.1371/journal.pcbi.1009558

**Published:** 2021-11-02

**Authors:** Eilam Goldenberg Leleo, Idan Segev

**Affiliations:** 1 The Edmond and Lily Safra Center for Brain Sciences, the Hebrew University of Jerusalem, Jerusalem, Israel; 2 Department of Neurobiology, the Hebrew University of Jerusalem, Jerusalem, Israel; National Research Council, ITALY

## Abstract

The output of neocortical layer 5 pyramidal cells (L5PCs) is expressed by a train of single spikes with intermittent bursts of multiple spikes at high frequencies. The bursts are the result of nonlinear dendritic properties, including Na^+^, Ca^2+^, and NMDA spikes, that interact with the ~10,000 synapses impinging on the neuron’s dendrites. Output spike bursts are thought to implement key dendritic computations, such as coincidence detection of bottom-up inputs (arriving mostly at the basal tree) and top-down inputs (arriving mostly at the apical tree). In this study we used a detailed nonlinear model of L5PC receiving excitatory and inhibitory synaptic inputs to explore the conditions for generating bursts and for modulating their properties. We established the excitatory input conditions on the basal versus the apical tree that favor burst and show that there are two distinct types of bursts. Bursts consisting of 3 or more spikes firing at < 200 Hz, which are generated by stronger excitatory input to the basal versus the apical tree, and bursts of ~2-spikes at ~250 Hz, generated by prominent apical tuft excitation. Localized and well-timed dendritic inhibition on the apical tree differentially modulates Na^+^, Ca^2+^, and NMDA spikes and, consequently, finely controls the burst output. Finally, we explored the implications of different burst classes and respective dendritic inhibition for regulating synaptic plasticity.

## Author summary

The output of most neurons consists of stereotypical electrical pulses, or spikes. Some neurons generate sparse spikes intertwined with groups of spikes in close succession, called bursts. Previous research has implicated bursts in long-range transmission, dendritic computation, and plasticity. However, a detailed account of the spatiotemporal synaptic activation patterns that generate bursts, even in the principal cortical pyramidal neurons, is largely missing. Using experimentally constrained computational modelling we determined the conditions of synaptic activation patterns that induce burst firing in these cells. We found two distinct classes of bursts. They are distinguished by the number of active synapses, on either basal or apical part of the dendritic tree, required for burst initiation, and by the number and frequency of output spikes. We provide an analysis of how location and timing of dendritic inhibition finely edit somatic bursts and show the change in the strength of excitatory synapses during bursts following their dendritic inhibition. Overall, this work offers a deeper understanding of the origin of bursts, including a novel distinction between burst classes and characterization of how inhibition may shape bursts and their correlated synaptic plasticity.

## Introduction

Layer 5 pyramidal cells (L5PCs) are considered to be pivotal building blocks of the mammalian neocortex [1]. In rodents, these cells receive ~10,000 synaptic inputs over their dendritic surface, about 20% of them originate from the local microcircuit and the rest from external structures. The thalamus accounts for ~10% of the synapses and represents the bottom-up input, while other cortical regions send connections reflecting top-down feedback and inter-module crosstalk [2–4]. The outputs of thin-tufted L5a PCs project laterally to nearby pyramidal cells and to other cortical areas. Thick-tufted L5b PCs project uniquely to subcortical regions (e.g., the thalamus) [5,6]. Their key importance in processing information was already realized more than 100 years ago by Ramon y Cajal (1894) [7], who termed cortical pyramidal cells “psychic” neurons.

Pyramidal cells in the neocortex *in vivo* tend to fire spikes in a random, Poisson manner such that the timing of each action potential is independent of its predecessors [8]. However, occasionally these cells also fire a brief burst of a few spikes at high frequency [9]. This occurs more than expected by chance (see Methods) [10]. Specifically, deep layer 5–6 pyramidal cells tend to burst [11,12], and thick-tufted layer-5b pyramidal cells show both tonic and burst firing intermingled [13].

It was shown that spike bursts convey different information about stimuli compared to isolated spikes, or else serve some other specific function. In the primary visual cortex (V1) during drifting-gratings stimuli, spike bursts in putative pyramidal neurons were tuned to the spatial frequency and orientation of the grating, while isolated spikes were tuned to their contrast [14,15]. In the electric organ of weakly electric fish, single spikes encode self-position whereas bursts better represent communication with other individuals [16]. However, researchers continue to argue whether bursts generally encode different features than single-APs [17] or only sharpen the tuning (for review see [18]).

Diverging lines of research probe burst involvement in additional functions other than feature-specific encoding. The BAC-firing coincidence detection mechanism [19,20] employs burst firing to associate the activity of several presynaptic neurons impinging on different parts of the dendrite. Bursting also gives rise to a substantial increase in vesicle release probability at the synapse [21] and promotes switching between various states during sleep [22]. Bursts could allow for data multiplexing [23,24], emphasize selective responses and propagate selective inputs [25]. Another debate still stands, whether more information is conveyed by the number of spikes in a burst [26,27] or by their firing rate [28]. Other approaches probe burst relevance to plasticity, showing that pairing L5PC excitatory post-synaptic potentials (EPSPs) with bursts or with a single spike led to long-term depression (LTD) versus potentiation (LTP) [29].

At the biophysical mechanistic level, Larkum, Zhu and Sakmann (1999) [30] were the first to demonstrate that bursting of cortical L5 pyramidal cells implements coincidence detection between perisomatic and tuft excitation (see also [20,31,32]). The basic *in vitro* procedure they used includes dual patch-clamp recordings targeting the soma and the main apical bifurcation (or ‘nexus’). In this paradigm, a backpropagating somatic action potential (bAP) lowers the threshold for a Ca^2+^ spike in the apical tree and, consequently, may lead to the generation of a somatic spike burst. They termed this phenomenon backpropagation activated Ca^2+^ spike firing (BAC firing). Many replicated and expanded their findings, including in some elaborate neuron models [33,34]. Without a bAP, dendritic input required for Ca^2+^ spike generation is much stronger and produces less somatic spikes [30]. The lowest threshold for burst generation was found when tuft activation followed the somatic action potential by ~5 ms.

There are several outstanding questions regarding the conditions for the initiation of bursts and the criteria for manipulating their characteristics. Details about the varied effects of dendritic inhibition on bursts are not yet clear. The necessity of the bAP for promoting Ca^2+^ spike firing and consequently bursting [30], is still questionable [35]. Furthermore, it is uncertain whether functional clusters of adjacent and temporally correlated inputs (whose existence was recently debated) [36,37] are sufficient for generating bursts?

The present work aims primarily to test various timing and location conditions of (basal and apical) excitatory synapses and of dendritic inhibition, for their effect on the initiation and control of somatic bursts. Towards this end, we employed the model built by Hay et al. [33], that utilized an automated feature-based parameter tuning to faithfully replicate both dendritic Ca^2+^ and perisomatic Na^+^ electrogenesis, and the interaction between these two spiking regions (i.e., BAC-firing). Shai et al. [38] employed this model to show how basal and tuft synapse numbers control high frequency bursting. This coincidence detection mechanism depends on voltage gated Ca^2+^ channels (VGCC), is approximated by a composite sigmoidal function, and can create orientation tuning. Our experiments introduce additional critical parameters that control this bursting mechanism, including dendritic inhibition, and suggest the involvement of bursts in Ca^2+^-dependent long-term synaptic plasticity.

## Results

### Temporal characteristics of excitatory dendritic inputs for burst generation

**Fig 1:**
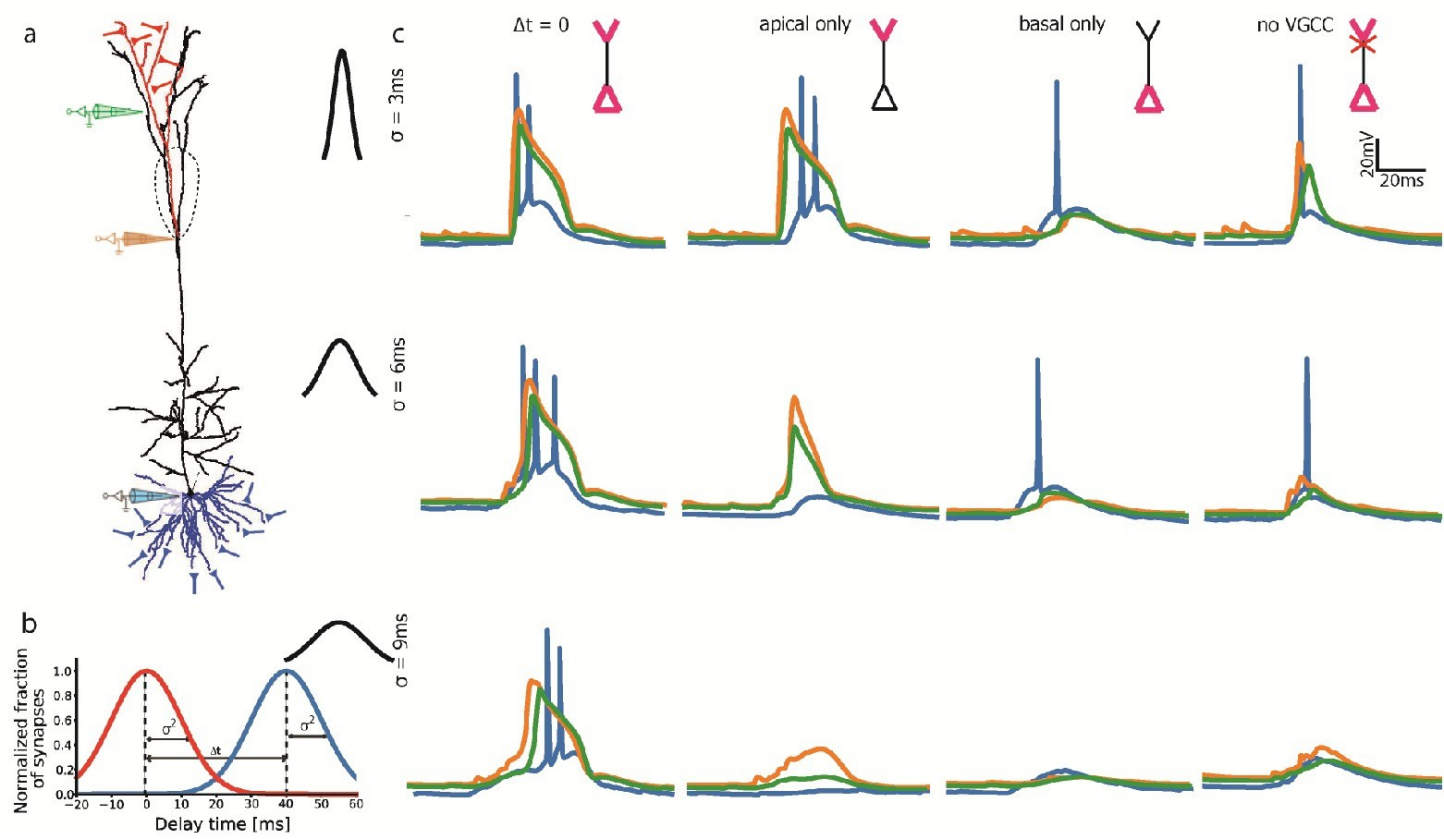
Burst initiation by basal and tuft input distributed in time. **a.** Model L5PC, schematically showing 50 basal (blue) and 30 apical tuft (red) excitatory synaptic locations (drawn from uniform distribution over the appropriate colored region). Electrodes are shown for corresponding voltage traces in **c**. **b.** Temporal distribution of input. Activation time is drawn from two tree-specific (same colors as **a**) normal distributions with equal variance and mean (unless noted otherwise). **c.** Voltage traces showing outcome of input to basal/tuft/both trees, with three σ values and no-VGCC control. Coincidental input to both trees results in burst firing for all σ’s. Tuft activation alone creates a burst for small σ, a dendritic spike for intermediate and nothing for high (equivalent to a lower synapse number). Basal activation results in a single AP at most, and removing VGCC returns all to one spike.

Spiking output of a cell depends critically on the number of activated synapses and their temporal correlation. To examine timing constraints on synaptic inputs for burst generation, we simulate a L5 PC model (Fig 1**A**; [33] and see **Methods**), and look at the response as we vary activation times of a fixed suprathreshold number of synaptic activations (Fig 1**B**). The synapses, which incorporated AMPA- and NMDA-dependent conductances (see **Methods**) were uniformly distributed on the entire basal dendrite (blue region in Fig 1**A**), and on 25% of the apical tuft (750 μm continuous stretch, red region in Fig 1**A**). Changing the percentage of the apical dendrite on which we distribute synapses promoted fewer bursts while the same manipulation in the basal tree did not alter it (S1 Fig, and **Methods**). We first examined the minimal number of excitatory synapses over these dendritic subtrees that, when activated synchronously, elicit a burst of somatic Na^+^ spikes. We found a threshold for synchronous activation of all synapses with a peak AMPA and NMDA conductance of *g*_*max*_ = 0.4 nS per synapse, at 50 ± 20 basal and 30 ± 10 apical tuft synapses.

Next, we explored the effect of the activation time of the synaptic input on burst generation. We randomly selected activation times for basal and apical synapses from two normal distributions of identical standard deviation σ (Fig 1**B**). Typical voltage traces for different σ’s and activation of synapses at either or both apical and basal dendrites are shown in Fig 1**C**. Increasing σ values from 0 (instantaneous) to 3 ms did not change the output burst substantially (data supplied in repository). The somatic burst comprised two Na^+^ spikes that were associated with a prominent Ca^2+^ spike (blue and orange traces respectively, in Fig 1**C** top left).

The voltage measured in the distal tuft compartment (green in Fig 1**C**) follows closely the voltage measured in the nexus (orange traces) except when Ca^2+^ spiking is partially abolished. With apical activation only (second column from the left) a Ca^2+^ spike is still generated, followed by a burst. With basal activation alone (third column) only one somatic spike is generated. With activation of both dendrites but without a Ca^2+^ hotspot (rightmost column), again only one somatic spike is fired. When increasing σ further to 6 ms and 9 ms (middle and bottom left traces respectively, in Fig 1**C**), the burst first consisted of 3 spikes (due to decreased voltage saturation) and then only two, with the Ca^2+^ spike essentially intact. With apical excitation only, higher σ resulted in Ca^2+^ spikes with lower maximum voltage and no burst (center and bottom rows in Fig 1**C**). With basal only excitation or no VGCC it finally resulted with no somatic spike at all. We conclude that initiation of bursts occurs by either coincidental basal+tuft excitatory inputs, or by substantially strong excitatory input to the tuft alone.

**Fig 2:**
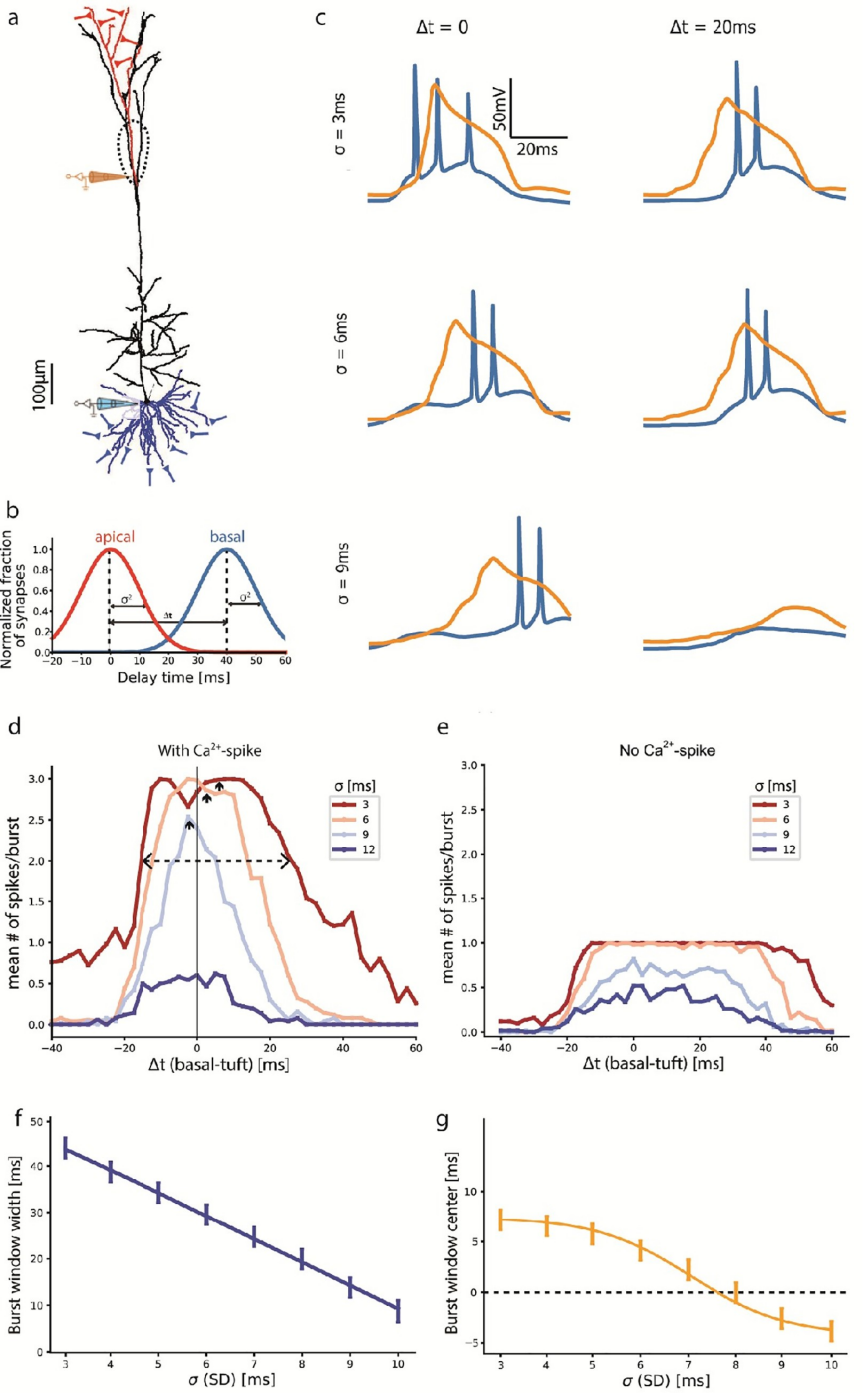
Temporal conditions for generating somatic spike bursts. **a.** Morphology of the modelled L5PC. Fifty excitatory synapses were distributed on the basal tree (blue synapses) and thirty on a subtree of the apical tuft (red synapses). Dashed line encircles the Ca^2+^ hotspot in the apical nexus. Blue and orange electrodes measure voltage traces shown in **c**. **b.** Two normal distributions of synaptic activation times for the basal and apical synapses are shown. The distributions have identical standard deviations, σ, and their means are shifted by Δt from each other. **c.** Voltage traces from the somatic and main apical bifurcation electrodes in **a** for a combination of σ and Δt values (Na^+^ spikes in blue and Ca^2+^ spike in orange). **d.** Mean number of somatic spikes per burst for a range of Δt values. Colored lines correspond to different σ values. The extent of Δt for generating a burst is reduced with increase in σ. Dashed line denotes curve width at 2 spikes per burst; arrows denote their centers. **e.** Same as **d** but without voltage-gated calcium channels, resulting in the absence of bursts. **f**, **g.** Width (**f**) and center (**g**) of the curves at two mean spikes per burst shown in **d.** Both width and mean reduce with increasing σ. Note in **g**, that the window’s center is positive (basal input following apical input) for smaller σ, and negative for larger σ.

In Fig 2 we increase the average delay Δt between the apical and basal synapses to 20 ms (right column in Fig 2**C**). With σ = 3 ms or 6 ms it resulted in a burst of only two late spikes, and the dendritic Ca^2+^ spike remained intact. With further increase in σ to 9 ms, the Ca^2+^ spike was abolished and thus the somatic firing also terminated (lower trace in right column, Fig 2**C**).

The summary graph (Fig 2**D**) of the mean number of spikes per burst for a range of Δt and σ values, shows that more spikes/burst are fired for small values of σ and Δt. The range of Δt with > 2 spikes per trial on average (above the dashed line in Fig 2**D**) defines the conditions for burst generation. Specifically, for σ = 3 ms (red line in Fig 2**D**), the range of Δt for burst generation spans 40 ms from Δt = -15 ms (basal synapses preceding tuft synapses) to Δt = +25 ms (tuft synapses then basal synapses). Interestingly, this window is slightly biased towards Δt > 0 (tuft preceding basal activation), as evident from the center value marked by a small upwards arrow in Fig 2**D**. Increasing σ decreases the number of spikes per burst and the range of Δt giving rise to a burst (Fig 2**C** and 2**D**). For σ = 9 ms, the Δt window for burst generation narrows linearly to 13 ms (from Δt = -8 ms to +5 ms; Fig 2**F**). At this high σ, Δt values for burst generation are centered near -5 ms (basal input before tuft), which was found experimentally to have the minimum threshold of current injection for burst generation (Fig 2**D** and 2**G** as in [30]). At even higher σ = 12 ms, the mean number of spikes per burst dropped to 0.5 and bursting vanished (dark blue in Fig 2**D**). For control we ran this experiment again without voltage-gated Ca^2+^ channels at the Ca^2+^ hotspot. This condition results with 1-spike maximum and no bursting, as there is no Ca^2+^ spike to boost the tuft input (Fig 2**E**, compare to Fig 2**D**).

Stretching synaptic activation times using σ allows a proxy for testing lower input intensities and their requirements of tuft-to-basal delay for burst initiation. Increasing σ, monotonically decreased both the Δt range for burst generation and the Δt-window center (small upwards pointing arrows in Fig 2**D**). Increasing σ shifted the center from positive delays at low values (tuft input preceding the basal input) to small negative delays at larger values (Fig 2**G**). We conclude that broadening the timing of synaptic activation narrows the tuft-to-basal delay window for burst generation (Fig 2**D** and 2**F**) and shifts the preferred order of activation from tuft-then-basal to basal-then-tuft (Fig 2**D** and 2**G**).

Ca^2+^ spikes are considered to be all-or-none signals, yet their duration may be modified, for example by the excitatory synaptic input that can prolong them or by inhibitory synapses that might cut them short. However, our traces show no substantial difference in Ca^2+^ spike duration for the activation of excitatory synapses at the range of σ = 3 and 9 ms (Figs 1**B** and 2**B**), nor for σ = 0 (data supplied in repository). A more systematic study is required to examine the correlation between the duration of the Ca^2+^ spike and the number and frequency of somatic Na^+^ spikes in a burst; this is beyond the scope of the present study.

To verify the mechanisms for burst generation, we compared our model with the established backpropagation-activated Ca^2+^ spike firing paradigm (BAC) [30] in Fig 1. In this paradigm, a backpropagating somatic AP lowers the threshold for an apical Ca^2+^ spike and a burst. A somatic depolarizing current injection results in a single somatic AP. This AP backpropagates up to the apical tuft, where, if it meets a subthreshold current injection, they may together generate a dendritic Ca^2+^ spike and consequently a burst consisting of 3 APs at the soma. The somatic and dendritic (“nexus” main apical bifurcation) voltage traces in Fig 1**C** show responses to synaptic activations with variable σ at either or both basal and tuft dendritic trees. Our model reproduces BAC-firing using conductance-based synaptic activation alone on the dendritic trees (Fig 1**C** middle-row), instead of the original experimental and more artificial current injection (see [33]). For both the original clamp experiment and our simulation, stronger tuft input without perisomatic activation (achieved here by decreasing σ; Fig 1**C** bottom) generates a Ca^2+^spike (orange) and a burst of only two APs (blue; Fig 1**C** top-center).

To check for burst dependence on voltage-gated Ca^2+^ channels (VGCC), we set their conductance to zero at the Ca^2+^ hotspot and noticed that all spikes disappeared, except for a single somatic one (Figs 1**C** and 2**E** right). Thus, we confirmed that low σ tuft input, combined with a somatic AP, creates a burst only via VGCC. Preserving VGCC and increasing σ to 9 ms caused tuft synapses to initiate a subthreshold voltage plateau, and prevented perisomatic synapses from generating an AP. Coincidental activation of the synapses on both trees may still elicit a full-blown Ca^2+^ spike and a somatic burst (Fig 1**C** bottom).

### Spatial input conditions and temporal burst characteristics

**Fig 3:**
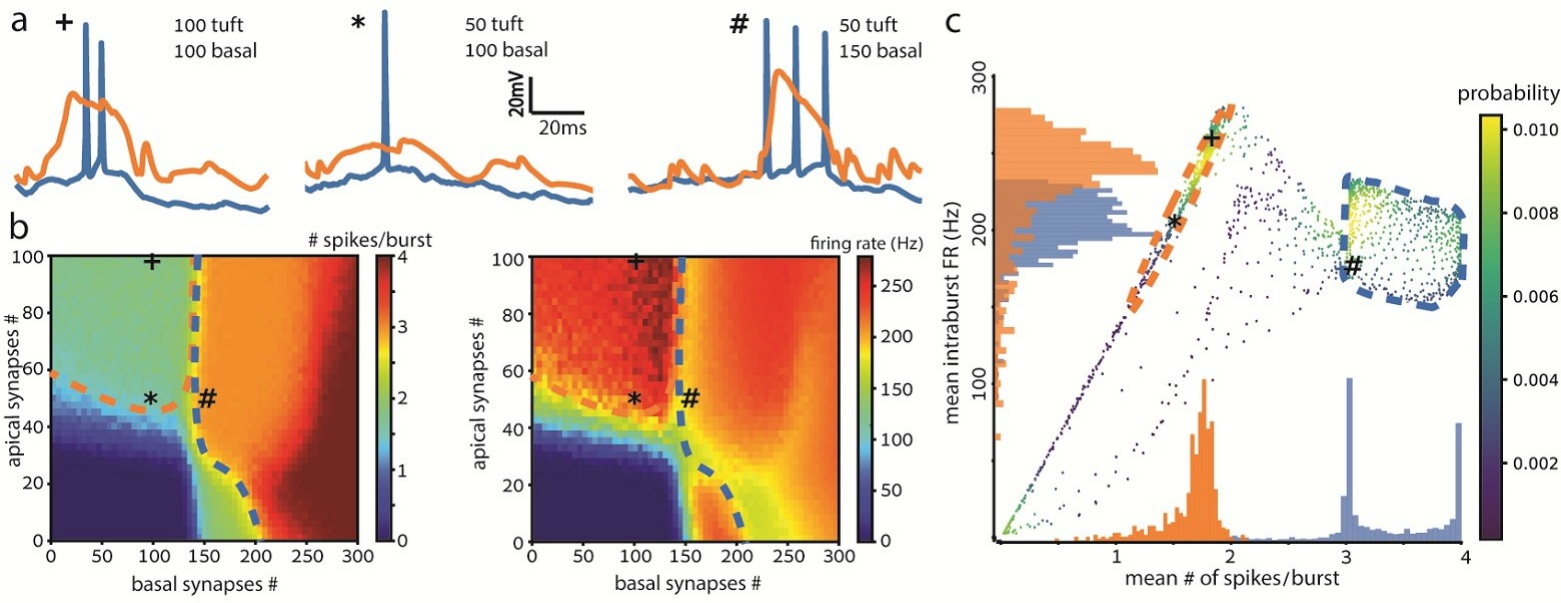
Two burst classes revealed based on the number of spikes per burst and intra-burst firing rate. **a.** Example somatic (blue) and dendritic (orange) voltage traces as in Fig 2 for the 3 cases of basal and tuft number of excitatory synapses: 100 and 100 (left), 100 and 50 (center), 150 and 50 (right). **b.** Heat maps of the mean # of spikes (left) and the intra-burst firing rate (right) for a range of numbers of basal and tuft synapses. The bursting threshold (> 2 spike/burst blue-green transition) is 40–60 tuft synapses or 140–150 basal synapses. Dashed lines denote smoothed class borders. Bursts with 3–4 spikes appear only for strong basal input (within the green dashed lines). Apical tuft input above threshold results in shorter bursts of 2 spikes and higher rates (within the purple dashed lines). **c.** Scatter plot for each combination of synapses (x-y coordinates) in **b**, sorted by the mean # of spikes per burst (left frame) and the intra-burst firing rate (right frame). Each value in right-hand heat map is plotted with its corresponding value in the left-hand heat map. The color of each point in **c** represents the density of data points with the same values, equivalent to their probability. The purple and green histograms along the x & y-axes correspond to the data in the 2 clusters encircled in purple and green, accordingly. Temporal input parameters were fixed at Δt = 0, σ = 10 ms.

We next explored the effects of the number of activated excitatory synapses that impinge on various parts of the dendritic tree, on burst generation (Fig 3). To do this we fixed the temporal parameters σ = 10 ms and Δt = 0, and separately manipulated the number of activated synapses on the basal tree and on a distal apical subtree (red subtree in Fig 1**A**). We used σ = 10 ms for a more realistic (dispersed) activation, and for better comparison with previous models that found some different results.

Fig 3**A** depicts three example voltage traces. When 100 basal and 100 tuft excitatory synapses were activated, a dendritic Ca^2+^ spike was initiated, accompanied by a burst of 2 somatic spikes (orange and blue respectively, Fig 3**A**, left). When the number of activated tuft synapses was reduced to 50, the burst disappeared and so did the dendritic Ca^2+^ spike (Fig 3**A**, center). Keeping the number of activated tuft synapses at 50 but increasing the activated basal excitatory synapses to 150 resulted in a burst of 3 spikes (with lower FR, see below) accompanied by a dendritic Ca^2+^ spike (Fig 3**A**, right).

Next, we measured the mean number of spikes per burst while independently varying the number of activated synapses impinging on both the basal dendritic tree and the apical tuft. Activating up to 50 apical and 140 basal synapses (with Δt = 0, σ = 10 ms) is mostly insufficient for generating a burst, and produces up to one spike (blue regions, Fig 3**B**; “*” indicates the example trace 3**a** center). 60 ± 10 apical synapses without basal synapses are sufficient for the generation of a burst of two spikes (top green region, Fig 3**B** left; “+” denotes the example trace, 3**a** left). A burst of two spikes is observed when 150–200 basal synapses (without apical synapses) are activated; increasing the number of basal synapses to 200–300 results in bursts of 3 or 4 spikes (orange and red regions, respectively, Fig 3**B** left; “#” denotes the example trace 3**a**, right). This finding is counterintuitive as tuft synapses are thought to be less potent in generating somatic spikes, due to the attenuation of their effect along the ~1 mm distance of the tuft dendrites from the soma. However, tuft synapses compensate more for this attenuation by NMDA spike generation, allowing burst-firing by fewer synapses (see **Discussion**).

The need to activate more synapses for burst generation in Fig 3 compared to Fig 2 is caused by the higher σ used in Fig 3; synaptic activation dispersed in time keeps local voltage from building up (by decaying towards the resting membrane potential), and specifically from promoting supralinear input summation by voltage-dependent NMDA receptors and Ca^2+^ channels (and thus their appropriate dendritic spikes too). What we found different than Shai et al. [38] was the addition of the basal-independent burst that we attribute to the smaller region we distributed synapses on the apical tuft, allowing stronger cooperation and dendritic spikes.

A supplementary analysis is shown in S2 Fig where the apical:basal ratio of the number of synapses is varied while the total number of activated synapses is fixed. For 50 or 100 total activated synapses, apical synapses alone promoted firing of one or two spikes, respectively, whereas the same numbers of basal synapses do not generate any spiking (red and grey lines in S2 Fig). 200 apical synapses alone generate bursts of 2 spikes, less spikes than with 200 basal synapses (2.5 spikes) or with 50 apical and 150 basal synapses (3 spikes/burst; blue line in S2 Fig).

Fig 3**B** right shows the intra-burst firing rate as a function of the number of basal and tuft synapses. An apical input of > 50 activated synapses, with a basal input of < 150 synapses, generates high-frequency bursts of ~250 Hz or, equivalently, inter spike interval (ISI) ≈ 4 ms (dark red area within purple dashed line in Fig 3**B** right), whereas higher basal input intensity of > 150 synapses results in bursts with lower rates of ~200 Hz (orange region, right of the green dashed line, Fig 3**B** right). Complementing Fig 3**B** left, our findings reveal a separation of bursts into discreet classes. One class of bursts contains a small number of spikes at high rates (2–3 spikes at ~250 Hz); this class is generated by the activation of many apical tuft synapses and mediated by an NMDA spike. To demonstrate that this burst class depends on NMDA-instigated Ca^2+^ spikes, we remove the NMDA conductance and show that burst firing no longer occurs from tuft input alone (S3**B** Fig and compare S3**A** Fig left and second left traces). We also compare the spikes per burst heatmap of Fig 3**B** (left; reproduced in S3**C** Fig) with that of AMPA-only synapses (S3**B** Fig), showing bursting only for stronger input (400 synapses) and that the NMDA dependent burst class is indeed abolished by setting g_NMDA_ = 0. The other class of bursts contains a larger number of spikes fired at lower rates (3–4 spikes at ~200 Hz) and is promoted by detection of coincidence of basal input with weak apical tuft activation.

Fig 3**C** is a scatter plot that combines the results of both plots in Fig 3**B**, and shows the mean number of spikes per burst (x-axis) versus the intra-burst firing rate (FR; y-axis). Namely, each point in Fig 3**C** represents the number of spikes per burst from Fig 3**B** left frame and its respective FR from Fig 3**B** right frame, for the same basal and apical synapse numbers. Three clusters emerged at regions of yellow and green points in Fig 3**C**, which are colored according to the probability of both spikes/burst and FR values. One cluster formed on the upper linear line in the scatterplot (purple dashed line, Fig 3**C**). It is characterized by a high FR of ~250 Hz and a mean of two spikes per burst. The second cluster appears as a rectangle to the right and is characterized by bursts of three or four spikes at a lower FR of ~200 Hz (green dashed line, Fig 3**C**). It is associated with coincidence detection of input to the basal dendrites and to the apical tuft, resembling BAC-firing as suggested by Larkum et al. [30]. The third cluster is located near the origin and reflects the case where no spikes were initiated, corresponding to the subthreshold blue areas in the two frames of Fig 3**B**.

A further quantification is attained by superimposed histograms of both burst classes (Fig 3**C**): in purple the high FR and two spikes class, and in green the lower FR and 3–4 spikes class (see **Methods**). These reveal clear separation in spike number and partial discrimination in intra-burst FR (histograms on x and y axes, respectively, Fig 3**C**). The scatterplot transforms the input-aligned picture of the heatmaps into a map of output burst characteristics showing each mean spikes-per-burst value with its corresponding FRs. Accompanied by the associated histograms, the scatterplot further elucidates the clear separation between the two burst classes.

### Editing bursts with inhibition

**Fig 4:**
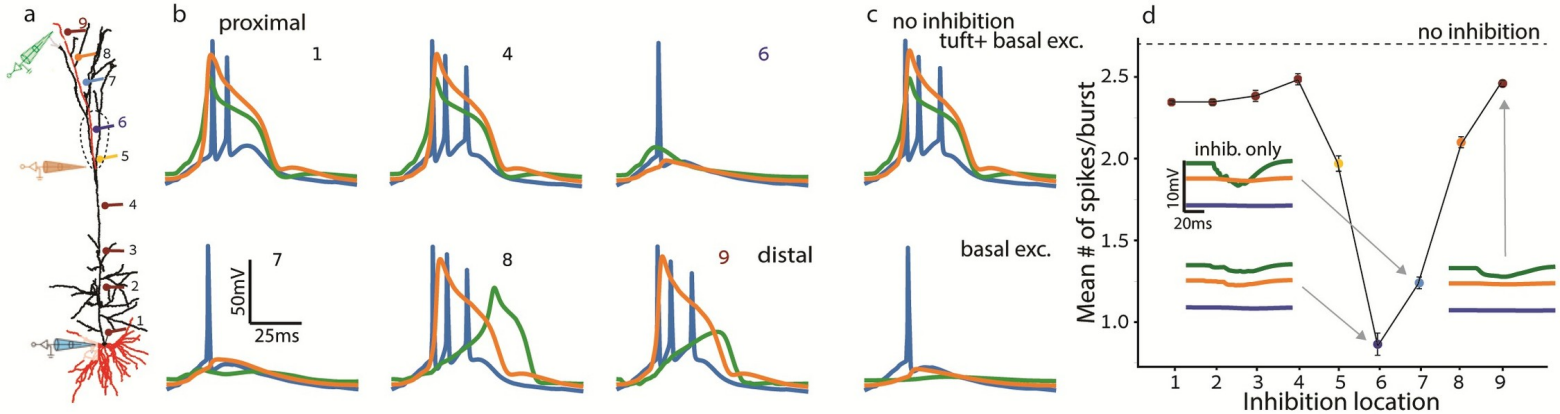
Inhibitory synapses edit the output burst differently depending on their dendritic location. The case of excitatory and inhibitory synapses restricted to a part of the apical tree. **a.** Model layer-5b thick-tufted pyramidal neuron as in Fig 1**A**. Electrodes indicate locations of voltage recording. Red branches in both basal and apical trees receive distributed excitatory synapses. **b.** Voltage traces from a somatic (blue) and two dendritic electrodes (orange and green) shown in **a**, during the activation of inhibition in a single locus (1–9) in the apical trunk (1,4) and apical tuft (6–9), indexed as that of the synaptic location in **a**. Proximal dendritic inhibition at location (1) suppresses the first Na^+^ spike (blue trace; compare to **c**, top trace) whereas inhibition at locations (6,7) abolishes the Ca^2+^ spike (orange) and NMDA spikes (green) and, consequently, the latter two spikes in the burst. Distal inhibition (8–9) only attenuates the terminal-branch NMDA spikes (green trace) but does not significantly affect the Ca^2+^ spike (orange trace) nor the somatic burst. **c.** Voltage traces without inhibition for basal and tuft excitation (top) and for basal-only excitation (bottom) for comparison. **d.** Mean # of spikes per burst as a function of location of inhibitory synapses (as in **a**). In all cases 70 basal and 40 tuft excitatory synapses were activated simultaneously (Δt = 0) with σ = 10 ms. For simulating inhibitory input, 20 inhibitory synapses were uniformly distributed up to 100 μm from each location (1–9) as marked in **a**; the peak conductance per inhibitory synapse was 1 nS (see **Methods**). Inset: Voltage traces of inhibition alone, in locations 6, 7 and 9, as pointed by the arrows.

A local modulation of pyramidal cell activity is provided by a spectrum of inhibitory interneuron types that are distributed over its dendritic tree [1]. We tested the sensitivity of burst generation to different locations of inhibitory synapses along the apical tree (Fig 4). Fig 4 shows a burst of 3-spikes, and the various edits that targeted inhibition may perform on it for different inhibition locations. We first selected a single continuous apical branch of length 600 μm (Fig 4**A**; compared to a random branching 750 μm in Fig 3) to receive excitatory synapses. The mean distance both between any two synapses, and from any synapse to the hotspot, is decreased by ~20% compared to Fig 3. The proximity and unbranched dendritic structure between synapses, and their vicinity, on average, to the Ca^2+^ hotspot better promotes supralinear summing of NMDA-mediated EPSPs and Ca^2+^ spike generation, thus generating more bursts and more spikes per burst. Inhibition was introduced by activating 20 GABA_A_ synapses at one of the numbered dendritic locations at each experimental condition (#1 - #9, Fig 4**A**). Varying the location of the activated inhibitory synapses produced the respective voltage traces in Fig 4**B**. The control case without inhibition is shown in Fig 4**C**.

When compared to the control case without inhibition (Fig 4**C**), we found that perisomatic apical trunk inhibition (at location #1) suppresses the first somatic spike (blue trace in Fig 4**B**1) but does not affect the dendritic Ca^2+^ spike (orange trace) nor the NMDA spike in the apical tuft (green trace). Distal trunk inhibition does not affect the Ca^2+^ spike, nor any somatic spikes (compare Fig 4**B** location #4 to Fig 4**C** top–no inhibition). Inhibition centered at the Ca^2+^ hotspot (#6) or adjacent to it, at about 100 μm distal (“off path”; #7) suppressed the Ca^2+^ spike and, consequently, the two latter somatic Na^+^ spikes, thus abolishing the burst. The impact of “off path” (rather than “on path”) inhibition on dendritic excitability was demonstrated and discussed by Gidon and Segev [39]. Finally, inhibition acting on the distal tuft hardly modulates Ca^2+^ or somatic Na^+^ spiking (compare locations #8 and #9 to Fig 4**C** top), but only alters local NMDA spikes (green traces).

The effect of the dendritic location of inhibitory synapses on burst generation is summarized in Fig 4**D**, showing the mean number of somatic spikes per burst for each inhibition locus (numbered as in Fig 4**A** and 4**B**). Clearly, the most effective inhibition on burst generation is located 200–400 μm distal to the apical nexus (points #6 and #7, respectively). Without excitation, distal inhibition hyperpolarized the distal dendritic terminal by ~2 mV (right inset trace). Inhibition in the hotspot did similarly, but also in the nexus (and the soma very slightly, bottom left trace). Inhibtion between the hotspot and the distal branches created the largest hyperpolarization of ~5 mV in the distal terminal (top left trace) which did not spread effectively beyond the tuft compartments. We varied the temporal order and separation (Δt_inh_) of excitation and inhibition and found no differential effect between dendritic locations, except for the effective time window width for inhibition. The inhibition with the largest effect (in location 6) disrupts spiking when activated even ±15 ms before or after excitation. Inhibition with a smaller effect (at locations #5, #8) contributes only when activated synchronously with excitation (data supplied in repository).

**Fig 5:**
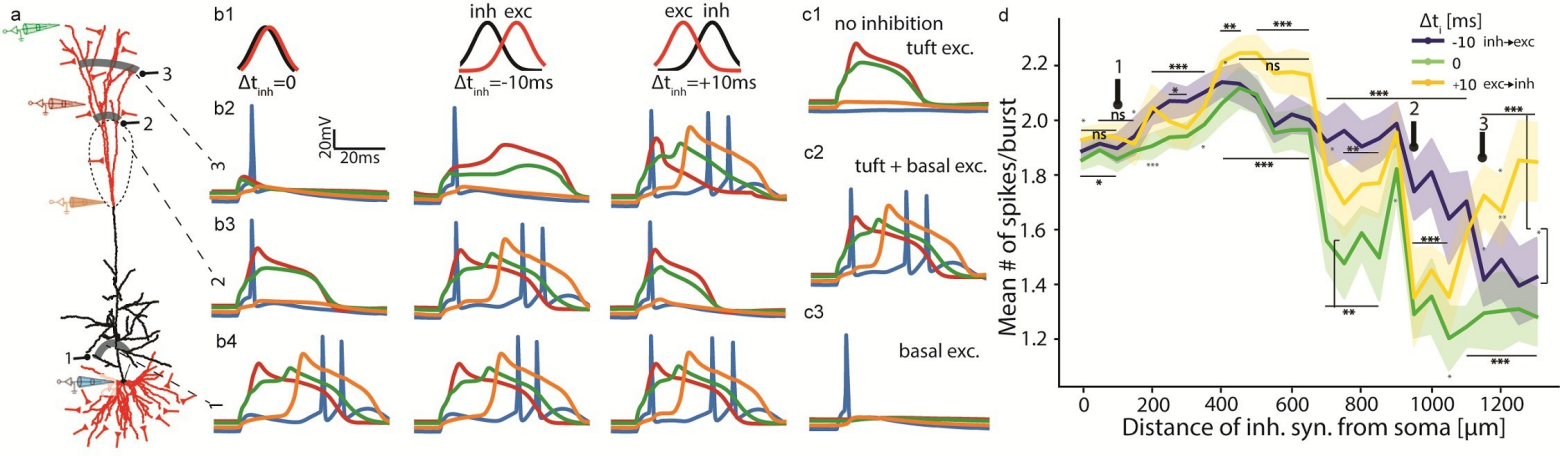
Impact of dendritic inhibition on burst generation. The case of whole apical tuft activation with dendritic inhibition distributed at strips of iso-distance from soma. **a.** Schematics showing the distribution of synapses. Inhibitory synapses at all locations of a fixed distance from the soma (a single numbered grey shaded strip). Synapses #1 at 100 μm from the soma on the oblique branches; synapses #2 at 950 μm from the soma on the intermediate apical tuft and synapses #3 at 1150 μm on the distal apical tuft. Electrodes correspond to colored voltage traces in **b**. **b1.** Normalized distribution of synaptic activation times separated for excitatory and inhibitory inputs, with different delays Δt_inh_ for the three columns shown. **b2-b4.** Voltage traces for inhibition at distances 1, 2 and 3, respectively. **c1-c3.** Control cases. **c1.** Forty excitatory synapses activated at the tuft triggers NMDA spikes but no dendritic Ca^+2^ spike nor somatic spikes. **c2.** Excitatory input to both basal (70) and tuft (40) dendrites generates an NMDA- (red/green) and a Ca^2+^ spike (orange) and, in turn, a somatic burst of Na^+^ spikes (blue). **c3.** Seventy excitatory synapses activated at basal dendrites generate a Na^+^ spike. **d.** Mean # of spikes in a burst as a function of the distance of inhibition from the soma. Lines correspond to different Δt_inh_ values as in **b**1. Shaded regions show standard deviation of 10 batch means (of 20 repetitions each; see **Methods**). P-values between Δt_inh_ = 0 and +10 ms are marked below green lines, Δt_inh_ = 0 and -10 ms above blue lines, and Δt_inh_ = ±10 ms above yellow lines. * p < 0.05, ** p < 0.01, *** p < 0.001. Unmarked are not significant (ns; p > 0.05). Excitatory synapses are as in Fig 4 (70 basal and 40 tuft), but distributed on the entire tuft, activated simultaneously (Δt = 0) with σ = 10 ms (see **Methods**). In each inhibitory stripe shown in **a**, 20 inhibitory synapses were activated in a 200 μm uniform distribution around all branches at each distance from the soma (1–3).

Next, we tested the case where the excitatory synapses were distributed over the entire apical tuft. Inhibitory synapses were distributed at all branches with a fixed distance from the soma (Fig 5**A**). The traces in Fig 5**B** show the influence of inhibition on somatic (blue) and dendritic tuft (orange red and green) voltage during burst generation. Each row relates to a single distance of inhibitory synapses: 100 μm from soma in bottom row, 950 μm in center, and 1150 μm in top. Each column depicts inhibition at a different timing condition: left Δt_inh_ = 0, center Δt_inh_ = -10 ms (inhibition before excitation), and right Δt_inh_ = +10 ms (inhibition after excitation).

Fig 5 shows that for 70 basal and 40 diffuse apical excitatory synapses, diverging inhibition achieves similar efficiency to the single-branch case (compare Fig 4**B** to Fig 5**B** left Δt_inh_ = 0, and compare Fig 4**D** to Fig 5**D** green): Perisomatic inhibition (location 1 in Fig 5**A**) suppresses the first spike of the burst (blue traces in Fig 5**B** bottom); above the Ca^2+^ hotspot (location 2) inhibition eliminates the Ca^2+^ spike (orange in Fig 5**B** center row; compare to Fig 5**C**2 –no inhibition), and at distal terminals (location 3) it attenuates close-by NMDA spikes whose absence disrupts the Ca^2+^ spike and burst firing (red and green traces in Fig 5**B** top; compare to Fig 5**C**1 –tuft excitation alone).

Surprisingly, this experiment reveals a novel inhibitory time dependence (Fig 5**B** columns and 5**D**), by which all the aforementioned inhibitory consequences appear at Δt_inh_ = 0 (Fig 5**B** left column), and at either Δt_inh_ = -10 ms (center column; inhibition before excitation), effective perisomatic (location #1) and distal inhibition (#3), or at Δt_inh_ = +10 ms (right column; inhibition after excitation), effective inhibition around the Ca^2+^ hotspot (#2). Only this last delayed inhibition effect resembles modelling and experimental results [40,41]. A comparison between inhibition effectiveness at various locations and under these three timing conditions (Fig 5**D**) exhibits preference for concurrent inhibition (Δt_inh_ = 0; green), then inhibition following excitation (Δt_inh_ = +10 ms; yellow) in intermediate tuft and preceding excitation (blue) in distal terminals.

The blue traces in Fig 5**B** show somatic spiking (intra-burst spike-count lower for both proximal trunk and tuft inhibition, see Fig 5**D**), and some additional depolarization from perisomatic (basal) current sources. Orange traces signify the Ca^2+^ spike at the hotspot (above the nexus) occurring for all tuft activations except nexus-proximal tuft inhibition (Fig 5**B** central row; decoupling NMDA spikes from the hotspot), either after voltage accumulation during extensive NMDA spikes (right) or concurrently with a somatic bAP (left). Red traces exhibit mid-branch NMDA spikes, and green their distal parallels, both eliminated by distal inhibition (Fig 5**B** top row). Each spike form and locus are manipulated by inhibition at a location ineffective for the other spike types, and only in part of the delay conditions.

Proceeding with whole-tuft excitation configuration, but slightly changing the balance of excitatory synapses between dendrites to 80 basal and 30 apical synapses (from 70 and 40 in Fig 5) creates a relatively “tuft-independent” burst generating scheme, where even strong (20 nS) tuft inhibition distributed on all branches of any one fixed distance from the soma (and nexus) does not prevent initiation of a Ca^2+^ spike and a burst. However, bursting generated in these conditions is affected by inhibition at the mid-upper apical trunk, decoupling the Ca^2+^ hotspot from its igniting bAP which did not happen in Fig 4 or Fig 5.

We determined optimal inhibition spatial extent by adjusting the uniform synaptic distribution to a variable portion of the apical tuft, the same as previously described for excitation, and presented together (S1 Fig). The optimal spatial dispersion for 20 inhibitory synapses with 1 nS peak conductance each (see **Methods**), which maximally decreases the number of spikes per burst, was 2.5–5% of the total apical dendritic length (5–10% of tuft length), or 185–370 μm. As illustrated in S1 Fig, this dispersion is at half or less the optimal extent of excitatory synapses (measured inversely, by finding maximal spikes per burst). We therefore fixed the dendritic length containing inhibition at 200 μm, i.e., a mean synaptic density of one inhibitory synapse in every ~10 μm, in line with experimental data [37], thus determining the specific locations most susceptible to inhibition of bursting.

### Impact of inhibiting bursts on the plasticity of excitatory synapses

**Fig 6:**
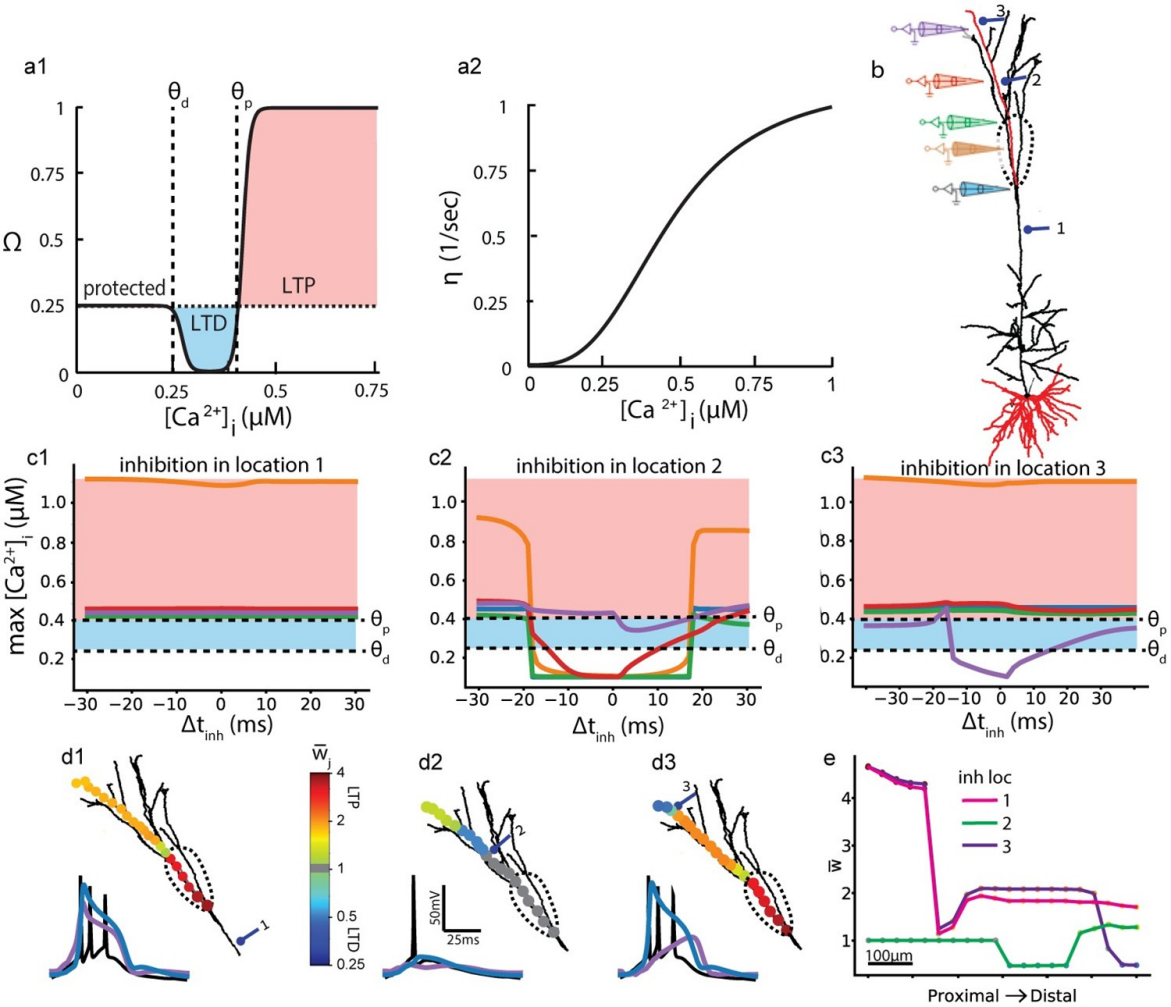
Impact of dendritic inhibition on somatic bursts and on plasticity of excitatory dendritic synapses. **a1.** Dependence of plasticity function, Ω, on intracellular Ca^2+^ concentration, [Ca^2+^]_i_. When [Ca^2+^]_i_ < θ_d_, the synaptic weights remain fixed (protected); for θ_d_ < [Ca^2+^]_i_ < θ_p_, synapses undergo long-term depression, LTD (blue) and for [Ca^2+^]_i_ > θ_p_ they undergo long-term potentiation, LTP (pink; see Eq (1) in **Methods**). **a2.** Learning rate, η, as a function of [Ca^2+^]_i_ (see Eq (2) in **Methods**). **b.** Model neuron and synaptic simulation parameters as in Fig 4**A**. 70 basal and 40 tuft excitatory synapses were activated (the “input”) on the red branches simultaneously (Δt = 0) with σ = 10 ms (see **Methods**). Electrodes and their respective color represent measurement locations of [Ca^2+^]_i_. Numbered synapses correspond to the mean locations of 20 inhibitory synapses with peak conductance 1 nS per synapse. Dashed line denotes Ca^2+^ hotspot. **c1-c3.** Maximal [Ca^2+^]_i_ along the apical dendrite at five locations (corresponding colored electrodes in **b**) following the input as a function of timing, Δt_inh_, between excitation and inhibition, for each inhibitory location (#1-#3 in **b**). Dashed lines mark threshold values, θ_p_ and θ_d_, as in **a**1. Blue shading—LTD; pink—LTP; white—protected. **d1.** Up: Twenty excitatory synapses are superimposed on the dendritic tree, color coded by their respective synaptic weights after activation of inhibition at location #1. The weights are normalized to their initial value (1). Changes are due to the plasticity protocol during 10 input repetitions for the case of Δt_inh_ = 0. Lower traces: Somatic (black); nexus Ca^2+^ spike (blue trace at respective blue electrode in **b**) and distal NMDA spike (purple at respective purple electrode in **b**) for inhibition location #1. **d2.** As in **d**1, with inhibition at location #2—burst and Ca^+2^ spike suppressed. **d3.** As in **d**1, with inhibition at location #3—distal NMDA spike suppressed, burst is unaffected. **e.** Changes in synaptic weights following the plasticity protocol as a function of synapse location along the tuft branch, for each of the three inhibitory locations.

Recent studies associate inhibition of bursts and of dendritic Ca^2+^ spikes with restriction of plasticity to specific connections for efficient learning [42–44]. To study how inhibition of bursts is associated with synaptic plasticity in the apical tuft, we utilized an established Ca^2+^-dependent plasticity model [45,46] (Eqs (1) and (2) in **Methods**, and Fig 6**A**1 and 6**A**21). Low Ca^2+^ concentration, [Ca^2+^]_i_, values result in “protected” (unchanged) efficacy of the excitatory synapses (Fig 6**A**), higher [Ca^2+^]_i_ produce long-term depression (LTD, Fig 6**A**1, blue), and even higher values elicit potentiation (LTP, Fig 6**A**1, pink) [45]. A Ca^2+^-dependent learning rate, η, (Fig 6**A**2) multiplies changes in efficacy (Eq (2) in **Methods**) to fit experimental findings. Using our model, we tested how L5PC excitatory synapses act under this plasticity rule, and explicitly how inhibition of dendritic Na^+^, Ca^2+^ and NMDA spikes (determined by location and Δt_inh_) controls the manifestation of this plasticity rule.

Fig 6**B** shows the modelled tree and the locations of the inhibitory synapses and measuring electrodes. Synaptic configuration is identical to that used in Fig 4. 70 basal and 40 tuft excitatory synapses were activated with (excitatory) Δt = 0 and σ = 10 ms. Fig 6**C**1–6**C**3 plots the maximal [Ca^2+^]_i_ at each electrode position (lines correspond to colored electrodes in Fig 6**B**) for a range of Δt_inh_ values (x-axis) with trunk (1), intermediate tuft (2) or distal (3) inhibition. Note that this maximum value of [Ca^2+^]_i_ does not fully represent whether an excitatory synapse at the location will be potentiated or depressed, as the time that [Ca^2+^]_i_ lasts at any given concentration will eventually determine the sign of plasticity. When [Ca^2+^]_i_ rises from baseline to LTP levels, it goes through LTD levels, so the time above the respective thresholds (together with the learning rate) determines the final synaptic weight (the plasticity sign and magnitude). I.e., if [Ca^2+^]_i_ crosses θ_d_ for a long duration but briefly reaches a maximum over θ_p_, then Fig 6**C** suggests LTP while actually the synapse would undergo LTD.

Fig 6**C**1 depicts the case in which the inhibition is proximal to the Ca^2+^ hotspot (at location #1) on the apical trunk. The maximum [Ca^2+^]_i_ at the hotspot is high and nearly invariant to inhibition timing at this inhibitory location (orange line in Fig 6**C**1), resulting with LTP for all Δt_inh_ values for synapses at the hotspot. The maximum [Ca^2+^]_i_ at the more distal locations is lower but above the potentiation threshold θ_p_, so weaker LTP is observed there (Fig 6**C**1). Fig 6**D**1 depicts the same case of proximal inhibition, specifically for Δt_inh_ = 0, by showing superimposed excitatory synapses on the dendritic tree, color coded by their respective synaptic weights after plasticity, normalized to initial values. Ten input repetitions were introduced in succession (but nonoverlapping) for a magnified effect on synaptic weights. For this proximal inhibition at location #1 the somatic burst is unaffected and therefore so is the Ca^+2^ spike at the hotspot (blue trace) and the distal NMDA spike (purple trace).

Fig 6**C**2 and 6**D**2 depicts the case in which the inhibition is 100 μm distal to the Ca^2+^ hotspot (at location #2). Well-timed inhibition (|dt_inh_|< 17 ms) at this location suppressed dendritic spikes, allowing Ca^2+^ influx only into the distal tuft (purple line in Fig 6**C**2). Without closely timed inhibition each Ca^2+^ spike promotes tuft-wide LTP. This observation suggests that any circuit that elicits frequent Ca^2+^ spikes and bursts must also incorporate some stabilizing inhibitory synapses to avoid uncontrolled potentiation. With inhibition activated in this location, the synapses proximal to inhibition including the hotspot are “protected”, and distal to it undergo LTD; only for the large positive or negative Δt_inh_ values (|Δt_inh_| > 17 ms) do synapses undergo LTP (Fig 6**C**2). At the distal dendritic locations (indicated by the red and purple electrodes in Fig 6**B**), the [Ca^2+^]_i_ (corresponding red and purple lines in Fig 6**C**2) is more sensitive to the timing of inhibition. This may be explained by the fact that the voltage response is all-or-none in the hotspot (blue inset traces in Fig 6**D**), while the distal branches (purple) may exhibit more variable levels of [Ca^2+^]_I_ due to the synergistic interplay between NMDA current and VGCCs voltage dependence. For this inhibition location for Δt_inh_ = 0, the somatic burst is suppressed and so are the Ca^2+^ spike at the hotspot and the distal NMDA spike (Fig 6**D**2). This severe dendritic voltage attenuation will lower the maximum distal [Ca^2+^]_i_ only slightly if active after excitation (Fig 6**C**2 purple line, similarly to NMDA spike suppression in [40]), but enough to switch between LTP and LTD.

Finally, Fig 6**C**3 and 6**D**3 depict the case in which the inhibition is at distal location #3. [Ca^2+^]_i_ levels are quite similar to inhibition in location #1, except for sites distal to inhibition where [Ca^2+^]_i_ is notably dampened (purple line in Fig 6**C**3). For distal inhibition the proximal synapses undergo LTP (as without inhibition) whereas synapses distal to the inhibition undergo LTD (Fig 6**D**3). The somatic burst and the Ca^2+^ spike are unaffected whereas the distal NMDA spike is attenuated severely by inhibition activated before excitation, shunting the backpropagating Ca^2+^ spike (inset voltage traces in Fig 6**D**3).

A summary plot of the change in the efficacy of the excitatory synapses along the excited dendritic branch for each inhibition location and Δt_inh_ = 0 is presented in Fig 6**E**. Inhibition in locations #1 and #3 (pink and purple lines in Fig 6**E**, respectively) create similar plasticity (LTP) patterns. The patterns differ considerably only at the distal terminal, proximal inhibition allows LTP and distal inhibition promotes LTD. Inhibition in location #2 (green line in Fig 6**E**) suppresses almost all plasticity, allowing mostly LTD in synapses distal to it. Note that the synaptic efficacies following the plasticity protocol are highly sensitive to the choice of potentiation and depression threshold (θ_p,d_) values for plasticity. In Fig 6 we fixed the thresholds at values conforming with previous modelling studies, but we provide supplementary results varying θ_p,d_ (S4 Fig).

We briefly summarize the key results of this section by noting that (1) inhibition located at the hotspot or immediately distal to it suppressed the Ca^2+^ spike and the branch-wide LTP; (2) inhibition of local NMDA spikes controls branch-specific plasticity by transforming LTP to local LTD or to the protected regime, and (3) following the association of bursts, Ca^2+^ spikes and inhibition with plasticity (see **Discussion**), we find a correlation between inhibition of bursts and limiting plasticity. The last point is in line with recent experimental studies, showing that limiting plasticity to specific connections relies on inhibition of bursts [42–44].

Finally, we tested for diverse outcomes of plasticity by the different burst classes and their corresponding inhibition, by utilizing the same Ca^2+^-dependent plasticity rule with whole-tuft excitation as in Fig 5**A**. We associate this scenario to “coincidence bursts”, in contrast to bursts resulting from putative NMDA spikes described in Figs 4 and 6. Full tuft excitation was mainly associated with low [Ca^2+^]_i_ outside the hotspot, meaning minor plasticity effects beyond LTP at the hotspot in the burst-suppressed dendritic tuft (S5 Fig). With these final results, our plasticity findings ultimately connect temporal (Figs 1 and 2) and spatial (S1 Fig) conditions for bursting with class separation (Fig 3), now evidently suggesting a functional basis (NMDA-burst dependent plasticity), through differential inhibition (Figs 4 and 5) and an implementation for restricting and tuning Ca^2+^-dependent plasticity (Figs 6 and S5).

## Discussion

Several studies have demonstrated the importance of spike bursts in pyramidal neurons in neural coding schemes [47], plasticity [48,49], and information flow [20,21]. Our understanding of the burst phenomenon as well as of dendritic Ca^2+^ and NMDA spikes mechanistically was well-established using simplified neuron models, dynamical systems and phase plane analysis [40,50–52]. In the present study, rather than simplifying the model in order to make it analytically tractable, we used the full nonlinear complexity of the modeled neuron together with detailed simulations to study the connection between the various dendritic spikes and spatiotemporal excitatory and inhibitory synaptic activation and somatic output bursts.

### Excitatory conditions for burst generation: Two types of bursts

We showed that somatic bursts arise either from a bidirectional stream of excitatory inputs combining basal and tuft synapses, or by generation of a local dendritic voltage plateau due to clustered excitatory input to the apical tuft alone (Figs 1 and S1). Bursts that result from a coincidence of basal and apical synaptic activation consist of 3–4 spikes fired at a frequency of ~200 Hz (Fig 3), whereas the activation of > 30 excitatory synapses on a length of 350 μm– 750 μm of the dendritic tuft (S1 Fig) generates NMDA spikes at several apical loci [53] initiating a Ca^2+^ spike at the main tuft and a high-frequency burst of ~2 spikes. The number of tuft synapses required for burst generation is less than half that of basal synapses (Figs 3 and S3). This finding is counterintuitive, as tuft synapses are thought to be less potent in generating somatic spikes, due to steep voltage attenuation expected in a length of ~1 mm along the apical dendrite towards the soma. Several factors compensate for the attenuation and make tuft synapses more likely to than basal synapses initiate bursts: clustered distribution (S1 Fig), high excitability of thin tuft terminals [53], and proximity of tuft synapses to the Ca^2+^ hotspot which promotes generation of Ca^2+^ spikes. Further experiments should be conducted to explore whether these two burst types exist in cortical L5 pyramidal neurons, and to reveal the implications of the different types for neuronal computation and for plasticity-related processes (see below).

In agreement with Larkum et al. [30], we found similar conditions on the timing of synaptic activation for burst generation when basal-then-apical (Δt < 0) synapses are activated (Fig 2). However, by varying the standard deviation, σ, of the apical and basal synapses activation times (Fig 2) we found that, for small σ (< 10 ms) burst generation is also enabled in the reverse tuft-to-basal order of activation (0 ms < Δt < 30 ms; Fig 2). This tuft-to-basal order was found favorable for Ca^2+^-dependent LTP in spines, resembling the classical asymmetric spike-timing dependent plasticity rules (STDP) [54]. This resulted from the long timescales and boosting effect of NMDA potentials that benefited from the bAP’s arrival after initial voltage buildup at the apical dendrites. Removing NMDA conductance disabled burst generation with Δt > 10 ms (S3**D** Fig). This prediction still awaits experimental validation.

### Inhibitory control of bursts

Next, we examined how the location and timing of dendritic inhibition ‘edits’ the somatic bursts. To the best of our knowledge, this is the first direct theoretical study of this question. For excitation on a single tuft branch, perisomatic apical trunk inhibition (e.g., via basket cells) abolished the first somatic spike but left the dendritic spikes unchanged and allowed for burst initiation (Fig 4**B**1). Inhibition in or immediately distal to the Ca^2+^ hotspot (“off path” condition) [39] disrupted the Ca^2+^ spike and suppressed the somatic burst (Fig 4**B**6 and 4**B**7 and 4**D**), whereas more distal inhibition attenuated local NMDA spikes without affecting the burst (Fig 4**B**8 and 4**B**9).

A burst induced by whole-tuft excitation was suppressed by inhibition distributed at all branches of a fixed distance distal and adjacent to the hotspot, for Δt_inh_ ≥ 0 (inhibition following excitation; Fig 5). Δt_inh_ ≤ 0 is more effective in disrupting burst generation when inhibition is activated in distal locations, by suppressing NMDA spikes there. Contrary to our results, theoretical and experimental studies found the most effective timing of inhibition for suppression of NMDA spikes to be after excitation [40,41]. This is because they use basal activation and set the criterion for efficiency of inhibition as the reduction in the voltage integral, rather than the strong instantaneous onset of NMDA spikes that is crucial for burst formation. Overall, we showed that by controlling local dendritic excitability, in particular the NMDA and/or Ca^2+^ spikes, local dendritic inhibition can finely edit the output bursts.

### Effect of inhibition on dendritic plasticity: Relationship to burst control

Notably, intracellular Ca^2+^ concentration is implicated in long-term synaptic plasticity [55,56]. Utilizing the calcium-dependent synaptic plasticity model [45], our study showed that inhibition, during somatic bursts and correlated Ca^2+^ spikes, enables diverse plasticity modification maps. Due to well-located and timed dendritic inhibition, local Ca^2+^ concentration in dendrites could be finely tuned, resulting in nearby synapses undergoing LTP or LTD, or left ‘protected’ from plasticity (Fig 6). These effects of synaptic inhibition on local dendritic excitability (modifying local dendritic NMDA and Ca^2+^ spikes) and, consequently, on synaptic plasticity are correlated with the impact of dendritic inhibition on burst activity. It seems that dendritic inhibition might simultaneously control local synaptic plasticity and global somatic burst activity (see also [42]).

In the case of single-branch activation whole-tuft LTP is very robust to the location and timing of inhibition (Fig 6**D**1 and 6**D**3). Furthermore, different inhibitory locations generate varied plasticity maps: segmenting the branch to LTP and LTD (distal tuft inhibition; Fig 6**D**3), or to LTP, LTD and protection of weights (intermediate tuft inhibition; Fig 6**D**2). However, for the case of whole-tuft activation, all inhibition locations distal to the intermediate trunk with correct timing reduce the tree-wide LTP to highly localized LTD (trunk or distal inhibition; S5**C**1 and S5**C**3 Fig) or limited adjacent LTP (intermediate tuft inhibition; S5**C**2 Fig), making this burst class worse in producing extended LTP or diverse local plasticity.

We showed that pairing EPSPs in the tuft with somatic bursting generally produces LTP (Fig 6**D**1). This prediction is supported by several experimental results [35,57,58] but disagrees with others [29]. In another paper, Owen et al. [42] suggested a role for burst inhibition that is compatible with our findings: limiting excitatory synaptic plasticity for efficient and stimulus-specialized implementation of learning. That SOM inhibition of bursts leads to ineffective plasticity was shown in the hippocampus [48] but not directly and causally in the cortex.

### Related studies

In their theoretical study, Shai et al. [38] showed that the intraburst firing frequency is best approximated by a composite sigmoidal function of the number of basal, and apical synapses, with apical number modulating the threshold of basal number required for bursting. This simplification is challenged by our findings, because it doesn’t generalize to the new class of bursts generated by single tuft branch activation (Fig 3). Specifically, their results do not show bursting for tuft only input, even at high numbers of synapses. Our findings require additional nonlinearities to account first for NMDA spikes and then for the non-monotonic transition in frequency between burst classes. The non-monotony of intraburst frequency is expressed in the corresponding heatmap (Fig 3**B** right). First more synapses mean higher frequency, but then it means shifting to coincidence-burst with lower frequency (compare to Shai’s [38] Fig 4**A** left). This discrepancy arises from our clustering of the apical synapses compared to their whole-apical dendrite distribution, leading to stronger interactions (more spikes, S2 Fig) and a basal-independent Ca^2+^ spike (Fig 1**C** and top green region in Fig 3**B**). However, our findings support Shai’s [38] result of bursting with basal only input (Fig 3; see also [29]), challenging the notion that bursts depend on Ca^2+^ spike firing [59,60].

Combined with our burst classes findings, we predict that input patterns generating coincidence many-spikes high frequency bursts (in which suppression of plasticity is more common) will also activate local SOM+ interneurons, and they would supply feedback inhibition near Ca^2+^ hotspot to withhold excessive LTP [23]. This could be tested by recording SOM-L5PC pairs verified for feedback inhibitory connections, possibly with excitation of presynaptic axonal projections. Analyzing their activity during L5PC burst/BAC-firing and using long-term plasticity indicators (e.g., AMPA/NMDA ratios, spine sizes) would validate our predictions.

### Implications of burst control on perception

Larkum et al. [19,20]suggested that the BAC firing mechanism implements coincidence detection between bottom-up sensory stream and top-down context modulation. Other studies made even stronger claims implicating bursts to be involved, via BAC firing, with conscious perception [61], visual illusions [62] and attentional modulation of activity [63]. In view of our findings of two types of bursts, these effects could be reexamined, and the impact of dendritic inhibition of L5 pyramidal neurons on such high-level processing explored. Particularly, Takahashi et al. [6,64]showed that optogenetic stimulation of L5bPC dendritic tufts enhances tactile stimuli detection, whereas blocking these cells’ outputs to subcortical target regions suppressed stimuli detection. We predict that distal tuft inhibition (e.g., by feedback via Martinotti cells) will attenuate local dendritic excitability and, in turn, decouple between the bottom-up and top-down input streams and, thus, disable conscious perception. As overreaching as the claims mentioned above seem to be, it is exciting to connect biophysical mechanisms such as specific dendritic inhibition to our subjective experience.

## Methods

All simulations were run using NEURON 7.7 [65] and Python 2.7.16 (NumPy v1.16.2), initially on Windows/Linux PC, and for final plots on a parallel processing cluster unit.

Code and generated data for obtaining the results are available at github.com/EilamLeleo/burst.

### Model cell

We used the established compartmental model of rat thick-tufted L5bPC developed by Hay et al. [33], including modifications of voltage gated calcium channel densities as in Shai et al. (2015) [38] and I_h_ channel density distribution as in Labarrera et al. (2018) [66]. The two similarly functioning reconstructed morphologies were used to verify our findings (see Fig 6 in [33]), though plots were generated with the first for convenience and consistency. This model consists of 10 different active membrane conductances, internal Ca^2+^ dynamics and hundreds of compartments arranged in 4 main section types: somatic, axonal and apical and basal dendritic. Hay et al. used a genetic algorithm in a procedure called multiple-objective optimization (MOO) to create thousands of working models combining all participating conductances in the different section types, with values within some experimentally constrained range. The resulting parameters span a subspace of continuous ranges which recreates the physiological voltage measurements. The greatness of their work is that unlike other compartmental models, they succeeded in fitting perisomatic and dendritic electrogenesis, and their interaction (i.e., backpropagation and critical frequency).

Viewing our simulated voltage traces, we noted a biologically unrealistic amplitude and prolonged duration of after depolarization (ADP) in the somatic Na^+^ spikes. Scanning our parameter space, we decided to change a single maximal conductance variable, so as not to significantly affect our results and the main findings of previous publications–that of the somatic calcium-dependent potassium current (SKv3_1). The peak conductance value of the SK channel was thus increased by 1.5-fold compared to that of Hay et al. [33] such that somatic g_SK_ = 3380*1.5 = 5070 pS/μm^2^.

### Simulations

Electric activity of the neuron was simulated for 600 ms at each instantiation with simulation dt = 25 μs. Simulation temperature was 34°C as previously suggested [67], and initial voltage was -76 mV.

We save all simulation data to Python NumPy arrays, initializing simulation of any experiment with each parameter combination at 100/200 random synaptic instantiations using a parallel processing unit (cluster). Randomly drawn properties were both spatial–site of synapse impinging on the dendrite, and temporal–activation time, as described in the following Input Distributions subsection. For most extensive data summarizing plots, we save only inter-spike intervals (ISIs), for calculation of spikes per burst and firing rate.

### Synapse models

Excitatory synapse model chosen (ProbAMPANMDA2.mod), implemented by Ramaswamy et al. [68] and modified by Hay et al. [33], combines a fixed ratio (of equal weights) of fast-AMPA (decay time constant τ_AMPA_ = 1.7 ms) and slower-NMDA (decay τ_NMDA_ = 43 ms) ionotropic receptors. Reversal potential for both was e = 0 mV. V_rest_ = -80 mV. Peak conductance gmax was fixed at 0.4 nS for both AMPA- and NMDA-synaptic conductances. Contemporary studies use a higher decay τ_NMDA_ = 70 ms, which is likely to make our findings of different classes more pronounced, as more current will be recruited by NMDA spikes. In the AMPA-only control (S3 Fig) we exchange ProbAMPANMDA2.mod with ProbUDFsyn2.mod which includes the same AMPA mechanism and parameters, but not the NMDA parallels–effectively setting g_NMDA_ = 0.

Inhibitory synapse model (ProbGABAAB_EMS.mod) [68] was preserved to include fast-decaying GABA_A_ only (decay τ_GABA_A_ = 8 ms), by keeping GABA_AB_ ratio = 0. Reversal e_GABA_A_ = -80 mV; with peak synaptic conductance g_max_ = 1 nS.

### Input distributions

Synaptic locations and activation times were randomly drawn from spatially uniform and temporally normal distributions, separated for basal and apical (excitatory) populations, and for inhibitory. For simplicity and lack of additional preliminary findings, all temporal distributions of any single simulation are identical in variance.

We note three main types of input patterns by how they spread on the dendritic tree: basal, apical or both. Basal branches are plentiful, interact at the soma and are less prone to NMDA-spike generation. Of course, some may be active spontaneously on the same branch and would contribute to an NMDA-spike formation, and a few of those will allow swift somatic APs or a burst [69]. However, the apical tuft allows this more readily by having long thin branches that create NMDA-spikes by fewer synapses [70], and by the Ca^2+^ hotspot transforming these into prolonged depolarizations at the soma. This lower threshold also means uniform distribution of synapses on the tuft will cause a burst before it would on the basal tree. Nevertheless, a bAP from the soma will lower the threshold for Ca^2+^-spike firing by the tuft and a burst, so the main options for bursting are tuft alone or both tuft and basal. How feasible, abundant and distinguishable are both input types? We show they are very much so.

For generating Figs 1–3, excitatory synapses were scattered on the entire basal tree, and on a randomly drawn continuous 750 μm stretch of the apical dendritic tuft, which is about 1/10 of the entire apical length or 1/5 of the tuft—equivalent to a single offshoot of the tuft from nexus to all distal terminals.

Various values of σ were used in Figs 1 and 2 (σ = 3,6,9,12 ms) to examine the implications of temporal jitter of synaptic activation on burst output. In Fig 3 we used σ = 10 ms as a single round parameter to test activation of many synapses on the different dendrites. We chose this value both for a more realistic (dispersed) activation, and for comparison with Shai et al. [38], that obtained some different results.

The delay described by Larkum et al. [30] from somatic spiking to apical EPSP isn’t the same as from basal to tuft synaptic activation, but we note a ~3 ms delay of both–somatic spiking from mean basal activation and apical EPSP peak from tuft activation, so this equivalence is accounted for. Synaptic noise was introduced for in-vivo like state by input of the same magnitude, but uniformly distributed on the dendrites and in simulation-time (600 ms starting 100 ms before targeted synaptic activity). Results were insignificantly different (except for a minor reduction in apical synapse number threshold for bursting in Fig 3 from 50 to 40).

For plotting Figs 4–6, inhibitory synapses were introduced on the apical tree. In all three, they were manipulated by distance from soma (20 synapses in < 200 μm disparity). Temporal jitter was allowed with the same parameters as excitation, excitation on both trees were activated simultaneously (Δt = 0), and Δt_inh_ was introduced to separate between excitation to inhibition mean activation times. For Figs 4 and 6, the number of excitatory synapses was increased from 50 basal and 30 tuft in Fig 1 to 70 basal and 40 tuft synapses, owing to the higher bursting threshold at σ = 10 ms. Tuft excitation was restricted to a single continuous branch of length 600 μm from nexus to the most distal terminal, inhibition is only on the trunk or the same branch. In Fig 5 excitation is on the entire tuft, and inhibition is at a fixed distance, but in all oblique or tuft branches sharing this distance from the soma.

### Data analysis

Most initial and direct analysis of electrodynamics in the model L5PC was calculated online on Python after each run of NEURON. Gathering of all data in any single experiment for plotting results and drawing conclusions was generally executed manually.

### Spike detection

We set the spike detection somatic voltage threshold at 0, though it essentially does not differ by setting it at -20 mV or +20 mV, nor by detecting it from the voltage trace at the axon initial segment. Spike width did not exceed 2 ms, and minimal ISI was above 3 ms, so effectively the soma was instantly hyperpolarized below detection threshold right after spiking, thus allowing for the next to be detected even at high intraburst (between spikes in a burst) firing rates of ~300 Hz.

### Number of spikes and firing rate calculation

The definition of bursts relates to statistical deviation of firing rate from random Poisson firing. A cell is considered bursting if either the coefficient of variation (CV = standard deviation/mean) or the Fano factor (= variance/mean) of ISIs over some time interval, is higher than 1 (Poisson). Relying on mean firing rates of pyramidal cells, we allow ourselves to group spikes as part of a burst by their ISI alone, < 20 ms (> 50 Hz): A burst of > 50 Hz will occur by chance for a Poisson neuron with a high 5 Hz mean firing rate (FR) approximately once every 10^15^ seconds. So, we set ISI ≤ 20 ms as our burst-grouping interval, and we note that our generated cell intraburst FR exceeds 100 Hz significantly. If no two spikes arrive within 20 ms of one another, then we count only one spike.

By this criterion we count and average the number of spikes per burst (at each simulation instance), even if isolated spikes precede the burst. The Δt range in Fig 2**F** is defined by > 95% chance for bursting and shown in Fig 2**D** as > 2 spike per trial average. Firing rate in Fig 3**B** and 3**C** is the mean over all spikes of all bursts in the same parameter combination, taking isolated spikes as a 0 Hz burst. Measuring intraburst FR for < 2 mean spikes per burst seems peculiar, because at least 2 spikes are needed in order to measure it, still it proves useful to keep values not only from bursts. We tested for big variations inside a single burst, that would discredit our conclusions–a burst of one ISI at 4 ms and another at 6 ms is not firing at 200 Hz but rather in the 170–250 Hz range. We find no substantial (1.5-fold) differences occurring in over 5% of instances at any parameter combination.

### Generating plots

All voltage traces represent a single representative simulation. Input pattern was kept fixed for parameters not probed at the particular experiment, i.e., changes in activation times do not alter spatial locations of synapses in Figs 1 and 2, and variations in inhibition do not modify excitatory pattern in Figs 4–6. In contrast, each value on the summarizing graphs and heatmaps (in Fig 3) averages 100–1000 random spatiotemporal synaptic patterns, with matching distribution parameters. Error bars are generally missing from all graphs that plot more than a single line. In Fig 2**F** and 2**G** they reflect sampling resolution. In calculating mean spikes per burst, standard deviation values are large, as any mean close to half a whole spike would indicate > 0.5 standard deviation. This will not reflect the consistency of our measurement, so instead we use (in Figs 4**D** and 5**D**) the standard deviation of batch means (10, each from 20 repetitions). Hence, the values are very generalizable and will occlude a possible variation within the fixed-parameter data.

The scatterplot in Fig 3 transforms the input-aligned picture on the heatmaps, into a mapping of each mean spike per burst value with its corresponding FR (each point represents a spike # from Fig 3**B** left and its collocated FR to the right). There we directly assess their correlations, and cluster output parameters pairs to three dense regions, high-rate low-spike #, low-rate high-spike # and zero spikes. These clusters in-turn map to correlating histograms, showing both parameter ranges attributed to each kind, when separated by input synapse numbers: purple generated by > 40 tuft and < 120 basal synapses; green by > 150 basal and < 100 tuft synapses.

### Calcium dependent plasticity

The learning rule applied is summarized by the two equations below (Eqs (1) and (2)) and graphs (Fig 6**A**) shown. The equations define the plasticity function Ω and the resulting learning rule as the change in synaptic strength (w_j_). θ_p,d_ are concentration thresholds for potentiation and depression respectively (Fig 6**A**1 and 6**C**), and η the learning rate (Fig 6**A**2).


$$\Omega =0.25+\frac{e^{80({\left[{Ca}^{2+}\right]}_{i}-\theta_{p})}}{1+e^{80({\left[{Ca}^{2+}\right]}_{i}-\theta_{p})}}-\frac{0.25e^{80({\left[{Ca}^{2+}\right]}_{i}-\theta_{d})}}{1+e^{80({\left[{Ca}^{2+}\right]}_{i}-\theta_{d})}}$$ (1)

$${\dot{\omega }}_{j}=\eta ({\left[{Ca}^{2+}\right]}_{i})(\Omega ({\left[{Ca}^{2+}\right]}_{i})-\omega_{j})$$ (2)

Graphs are created for maximal [Ca^2+^]_i_ (concentrations) during the simulation at different inhibitory locations and times (Fig 6**C**). Threshold was slightly shifted from the previous studies [45,46] to θ_p_ = 0.4 μM, θ_d_ = 0.25 μM (originally 0.5 and 0.3), which creates a more variable and realistic range of phenomena (LTP/D & protected). To generate synaptic weight changes in Fig 6**D**, the duration over or under any threshold is multiplied by a learning factor chosen as ten repetitions for effect size, as physiological [Ca^2+^]_i_ measurements are difficult to control for in these relatively short simulations. Synapses on trees represent predicted weights after learning by this rule.

## Supporting information

S1 Appendix Additional burst control. (DOCX)

S2 Appendix “Off-path” inhibitory control of bursting. (DOCX)

S3 Appendix Complementary plasticity comparisons. (DOCX)

S1 Fig Spatial extent of synapses controls burst generation. Number of spikes generated as a function of percent of dendritic tree on which excitatory alone (red apical; blue basal, apical on 5%) or inhibitory (black; apical excitation on 10%) synapses are distributed. Excitation continues over 20% at a different scale. Dashed line: apical excitation with VGCC blocked. Excitatory conditions as in Figs 1 and 2 (σ = 9 ms, Δt = 0), inhibition as in Fig 4.
(TIF)

S2 Fig The number of spikes per burst with fixed total activations depends on apical:basal ratio. Left: heatmap of mean number of spikes per burst as a function of the number of activated synapses on the basal and apical trees, from Fig 3**B**. Overlaid are fixed total synapse number diagonal lines, whose profile is plotted to the right. Right: Mean number of spikes per burst, as a function of the ratio between apical and basal synapse number, for various total synapses, as plotted on the respective colored diagonals on the left. σ = 10 ms, Δt = 0.
(TIF)

S3 Fig Bursting is partially abolished by removing NMDA conductance. **a.** Somatic, nexus and distal tuft voltage traces (blue, orange and green, respectively), after activation of 30 tuft synapses (σ = 3 ms) with and without NMDA or distal VGCC. **b.** Heatmap of mean number of spikes per burst as a function of the number of activated synapses on the basal and apical trees, with AMPA-only synapses. The apical-only burst class vanishes with zero NMDA conductance. **c.** Same as **b** but as a control experiment with both AMPA and NMDA conductances (from Fig 2**B**). **d.** Mean number of somatic spikes per burst for a range of Δt values, without NMDA. Colored lines correspond to different σ values. **e.** Same as **d** but for control with NMDA (from Fig 2**D**). Note that the bursting window’s center is negative (basal input following apical input) without NMDA for small σ.
(TIF)

S4 Fig Ca^2+^ dependent plasticity patterns at different thresholds. [Ca^2+^]_i_ measured as in Fig 5**C**2 (intermediate inhibition location, Δt = 0) were used for calculating plasticity modifications as in Fig 5**C**2, for a range of threshold θ_p/d_ values. The heatmap represents synaptic weight after plastic modification, normalized to initial value and colored on a log_2_ scale (Red LTP, blue LTD, 0 –green protected). Modified excitatory synapse location is ordered on x-axis. LTP threshold θ_p_ on y-axis. LTD threshold θ_d_ is kept at a fixed ratio of 0.6 to θ_p_.
(TIF)

S5 Fig Ca^2+^-based plasticity of excitatory synapses during burst suppression in the whole-tuft case. **a.** Model neuron as in Fig 4**A**. Red branches are excited. Electrodes for [Ca^2+^]_i_ measurement as in Fig 5**A**. Inhibitory synapses at all locations of a fixed distance from the soma (a single numbered grey shaded strip). Dashed line denotes Ca^2+^ hotspot. **b1-3.** Maximal [Ca^2+^]_i_ along the apical dendrite at the three locations (colored electrodes in **b**) as a function of Δt_inh_ between excitation and inhibition for each inhibition location. Dashed lines mark thresholds, shadings the resulting change–LTP (red), LTD (blue). **c1-3.** Synaptic weights after repeated execution of the learning rule (Eq (1), **Methods**) for 10 input repetitions (60 seconds) and Δt_inh_ = -5 ms at each inhibition distance # (**a**,**b**). Twenty representative excitatory tuft synapses are color plotted by their respective weights: yellow—LTP (in 2), blue—LTD, and grey—protected.
(TIF)
