# Supplementary material for: Burst control: Synaptic conditions for burst generation in cortical layer 5 pyramidal neurons
Source: PLoS Comput Biol. 2021 Nov 2;17(11):e1009558. doi: 10.1371/journal.pcbi.1009558 (PMC8589150; doi:10.1371/journal.pcbi.1009558)
Supplement: S1 Appendix — (DOCX) [file pcbi.1009558.s001.docx]

#### S1 Appendix. Additional burst control

Input to the apical obliques was suggested to better couple input to the apical tuft with that to the perisomatic/basal compartments, which is beneficial for BAC firing [1]. Our simulations show no significant role for these synapses.

A different dendritic spiking mechanism than BAC firing is driven by the coactivation of clustered NMDA receptors in neighbor dendritic synapses. Having a longer time scale of activation (large time constant) than AMPA receptors, higher conductance values, and high penetration to Ca^2+^ [2], NMDA synapses may largely dictate the neuronal response. But in large pyramidal cells, some of these synapses are located on the far distal tuft, and hardly give rise to a somatic excitatory post-synaptic potential (EPSP) due to voltage attenuation along the elongated apical dendrites [2]. Therefore, what is their function?

A considerable number of activated tuft synapses, especially in conjunction with a bAP, may ignite a Ca^2+^ spike and a burst. Besides, NMDA synapses show voltage dependence, which enhances the coactivation of others nearby by relieving channel blocking Mg^2+^ ions. This dependence creates the NMDA spike, which requires less coactivated synapses in the tuft than in basal dendrites [3]. This phenomenon appears either isolated in a dendritic fragment of ~30-70 μm (focal) or in a global whole-tuft reach. Actually, researchers have claimed that NMDA spikes in the tuft directly and significantly affect somatic firing only by generating a Ca^2+^ spike [2,4]. We showed that activating some tens of AMPA/NMDA synapses on a moderate length of dendritic tuft (350-750 μm) creates extended NMDA spikes, initiating a Ca^2+^ spike (or decoupled from it by inhibition) and a burst of fewer spikes and higher rate than that of BAC-firing (Fig 2).

To better simulate natural input by decreasing temporal input correlations, we introduced a normal distribution for drawing activation times, and gradually increased its variance (σ^2^; Figs 1**b** and 2**b**). For examination of tuft-to-basal mean activation delay we separated the distribution in two of identical σ^2^ and different means, denoting their disparity Δt. This distribution form allows dependence on a variable separation between two distributions, as opposed to an overlap range only in uniform distributions. A supralinear sum of simultaneous synaptic activations would decrease if they were distributed over longer timescales (Fig 1**c**) due to reduced overlap. This dispersing is equivalent to decreasing the number of active synapses at a fixed time interval. Consequently, the dendritic event amplitude and spikes per burst, or bursting altogether, may diminish (Figs 1**c** and 2**c**). Raising σ values from 0 (instantaneous) to 3 ms did not change the output burst significantly.

Typical *in vivo* conditions may create stronger spatial correlations (i.e., clusters of neighbor synapses) and weaker temporal correlations (dispersed activation times). So, we continued by checking the optimal spatial extent of synapses in the tuft for bursting (S1 Fig). We predicted that only the tuft extent will have a noticeable effect because the basal dendrite requires more synaptic activations for NMDA spikes [3], affords less proximity to Ca^2+^ hotspot, and interactions between branches are obtained at the soma and not on dendrites. We found the highest burst probability and spikes per burst arise for synapses distributed over 350-750 μm of a continuous dendritic tuft length (S1 Fig, as illustrated in Fig 1**a**) which is optimal for supralinear summation and does not begin to saturate (by a few tens of synaptic activations) the membrane voltage. Synaptic activation confined to a smaller dendritic extent or spread over larger areas will decrease spike number.

In an experiment modifying the length of basal dendrite on which synapses are distributed we found no consequential difference on spikes per burst, suggesting less tendency for supralinear or regenerative events in basal dendrites vs. the apical tuft. One potential explanation for this result is that the basal dendrites include no active conductances (except for the voltage dependence of the NMDA receptors). Additionally, we ran separate conditions for different percentages of the apical dendrite on which we distributed synapses, this time while blocking the voltage-gated Ca^2+^ channels (VGCCs), and found that all percentage conditions result with a single spike (dashed line in S1 Fig). These findings support our choice of limiting spatial distribution of synapses on the apical tuft and not on the basal dendrite, and agrees with studies showing that basal branches are less prone to generate NMDA spikes [5].

In S2 Fig we plot spikes/burst graphs of fixed total number of synapses, varying the percentage distributed on the apical and basal trees. In S3**b** Fig we show that the NMDA dependent burst class is indeed abolished by setting gNMDA = 0. The resultant graph resembles Shai et al.'s [6] results with bursting by basal-only input, modulated by apical input. Without NMDA channels the number of synapses required for burst generation is much larger. This follows from the lower remaining conductance of AMPA-only synapses. However, whereas the number of basal synapses required doubles from 150 to ~400, the number of required tuft synapses increases from ~50 to 400 as well. This difference is due to the nonlinear boosting effect of the voltage-dependent NMDA receptor, generating a dendritic spike capable of initiating a burst even without prior spiking at the soma.

We attribute the new burst class in Fig 3 to generation of NMDA spikes in the distal apical tuft, leading in turn to a Ca^2+^ spike and a short burst of 2 spikes with high frequency. To verify our hypothesis, and to connect with related timing and inhibition results, we ran experiments described in Figs 2 and 3 without NMDA receptors. We found that the small density of distal VGCC aid in EPSP summation for Ca^2+^ spike and burst initiation (S3**a** Fig), that the novel burst class is indeed abolished by removing NMDA, and that bursting with Δt > 0 is absent too.

With AMPA and NMDA receptors (S3**a** Fig left), an NMDA spike is initiated first in the distal tuft (green), followed by a Ca^2+^ spike (orange) and a burst of 2 somatic APs (blue). Without NMDA (next trace to the right) only a big EPSP is measured in the distal tuft, and no Ca^2+^ spike or somatic APs are generated. When NMDA conductance is present but distal (to the hotspot) VGCCs are blocked, a faster decaying NMDA spike is generated and no Ca^2+^ or somatic spikes appear. Lastly, without NMDA, blocking distal VGCC does not alter the voltage response significantly.

We also demonstrate that by activating inhibitory synapses between the Ca^2+^ hotspot and the distal NMDA spike initiation segment, we can decouple the distal dendritic spike from the Ca^2+^ spike initiated in the hotspot (Fig 3**b**8 and Fig 4**b**, **c**).

#### References

1. Larkum ME. Top-down Dendritic Input Increases the Gain of Layer 5 Pyramidal Neurons. Cereb Cortex [Internet]. 2004 Apr 27;14(10):1059–70. Available from: https://academic.oup.com/cercor/article-lookup/doi/10.1093/cercor/bhh065

2. Polsky A, Mel B, Schiller J. Encoding and Decoding Bursts by NMDA Spikes in Basal Dendrites of Layer 5 Pyramidal Neurons. J Neurosci [Internet]. 2009 Sep 23;29(38):11891–903. Available from: http://www.jneurosci.org/cgi/doi/10.1523/JNEUROSCI.5250-08.2009

3. Poleg-Polsky A. Effects of Neural Morphology and Input Distribution on Synaptic Processing by Global and Focal NMDA-Spikes. Brown KS, editor. PLoS One [Internet]. 2015 Oct 13;10(10):e0140254. Available from: https://dx.plos.org/10.1371/journal.pone.0140254

4. Grienberger C, Chen X, Konnerth A. NMDA Receptor-Dependent Multidendrite Ca 2+ Spikes Required for Hippocampal Burst Firing In Vivo. Neuron [Internet]. 2014 Mar;81(6):1274–81. Available from: https://linkinghub.elsevier.com/retrieve/pii/S0896627314000191

5. Polsky A, Mel BW, Schiller J. Computational subunits in thin dendrites of pyramidal cells. Nat Neurosci [Internet]. 2004 Jun 23;7(6):621–7. Available from: http://www.nature.com/articles/nn1253

6. Shai AS, Anastassiou CA, Larkum ME, Koch C. Physiology of Layer 5 Pyramidal Neurons in Mouse Primary Visual Cortex: Coincidence Detection through Bursting. Sporns O, editor. PLOS Comput Biol [Internet]. 2015 Mar 13;11(3):e1004090. Available from: https://dx.plos.org/10.1371/journal.pcbi.1004090
