# Supplementary material for: Burst control: Synaptic conditions for burst generation in cortical layer 5 pyramidal neurons
Source: PLoS Comput Biol. 2021 Nov 2;17(11):e1009558. doi: 10.1371/journal.pcbi.1009558 (PMC8589150; doi:10.1371/journal.pcbi.1009558)
Supplement: S2 Appendix — (DOCX) [file pcbi.1009558.s002.docx]

#### S2 Appendix. “Off-path” inhibitory control of bursting

Inhibition at or close and distal to the Ca^2+^ hotspot (“off path”, similarly to [1]) disrupted the Ca^2+^ spike and burst from forming (Fig 3**b** and 3**c**). These results relate to previous works showing that distal inhibition (“off path”) is more efficient at terminating dendritic spikes than proximal inhibition (“on path”) [1]. They keep all excitatory input in a single point (“hotspot”) able of generating a Ca^2+^ spike, whereas we distributed the excitation, meaning that our “off path” is actually between the majority of excitatory synapses and the Ca^2+^ hotspot, rather than distal to them. Distal tuft inhibition still better attenuates nearby NMDA spikes but not the Ca^2+^ and Na^+^ spikes directly. Somatostatin-expressing (SOM) interneurons were found to innervate L5PC tuft [2], and within it the Ca^2+^ hotspot. Thus, we draw the conclusion that regulation of burst firing is amongst their core roles (similarly to [3,4]).

#### References

1. Gidon A, Segev I. Principles Governing the Operation of Synaptic Inhibition in Dendrites. Neuron [Internet]. 2012 Jul;75(2):330–41. Available from: https://linkinghub.elsevier.com/retrieve/pii/S0896627312004813

2. Wang Y, Toledo-Rodriguez M, Gupta A, Wu C, Silberberg G, Luo J, et al. Anatomical, physiological and molecular properties of Martinotti cells in the somatosensory cortex of the juvenile rat. J Physiol [Internet]. 2004 Nov;561(1):65–90. Available from: http://doi.wiley.com/10.1113/jphysiol.2004.073353

3. Royer S, Zemelman B V, Losonczy A, Kim J, Chance F, Magee JC, et al. Control of timing, rate and bursts of hippocampal place cells by dendritic and somatic inhibition. Nat Neurosci [Internet]. 2012 May 25;15(5):769–75. Available from: http://www.nature.com/articles/nn.3077

4. Goldberg JH, Lacefield CO, Yuste R. Global dendritic calcium spikes in mouse layer 5 low threshold spiking interneurones: implications for control of pyramidal cell bursting. J Physiol [Internet]. 2004 Jul;558(2):465–78. Available from: http://doi.wiley.com/10.1113/jphysiol.2004.064519
