# Supplementary material for: Burst control: Synaptic conditions for burst generation in cortical layer 5 pyramidal neurons
Source: PLoS Comput Biol. 2021 Nov 2;17(11):e1009558. doi: 10.1371/journal.pcbi.1009558 (PMC8589150; doi:10.1371/journal.pcbi.1009558)
Supplement: S3 Appendix — (DOCX) [file pcbi.1009558.s003.docx]

#### S3 Appendix. Complementary plasticity comparisons

Our previous results imply diverging outcomes for plasticity by the different burst classes and corresponding inhibition. We test this by applying the same Ca^2+^-dependent plasticity procedure, while using the whole-tuft excitation configuration as in Fig 4. The results are well in line with our hypothesis, as full tuft excitation is mainly associated with lower [Ca^2+^]_i_  than the single-branch case, which generally means reduced plasticity effects in the burst-suppressed dendritic tuft (S5 Fig). In response to bursting, without inhibition, our model produces strong LTP at the hotspot, and minute LTP throughout the apical tuft (on average, although some sites may show LTD). We defined inhibition distances at 500, 1100, and 1300 μm from the soma for comparison with the single-branch case (Fig 5). We remind that in this synaptic configuration inhibition is distributed in all branches at these distances, and note the difference from same distance indexes in Fig 4. Distal tuft inhibition (#2-#3) activated even 20 ms before or synced to excitation (as in Fig 4) drops [Ca^2+^]_i_ below the effective thresholds (S5**b**2, **b**3 Fig), resulting in protected synaptic efficacies, with the exception of the most distal branches which are depressed (S5**c**3 Fig) or slightly potentiated (S5**c**2 Fig).

Trunk inhibition (location #1 in S5**a** Fig) too shows suppression of the Ca^2+^ spike and burst, and minor distal LTD, but adheres to a highly specific timing restriction, limited to inhibition 5 ms prior to excitation (dip in orange and blue lines in S5**b**1 Fig, and see Fig 1) [1]. This timing supports both the bAP role in burst initiation (identifying our coincidence class), and vicinity to the bursting threshold, because no other delay suppressed bursting.

Such complex multilevel simulations as our plasticity experiments, presume many assumptions, some of which we did not tackle explicitly (dominance of [Ca^2+^]_i_ in plasticity, learning rule shape thresholds and saturation, dendritic shaft equivalence to spines, influx through VGCC and not NMDAR, no diffusion, etc.). Even so, it allows an elaborate demonstration, starting from the established effect of global dendritic Ca^2+^ spike that promotes LTP throughout the tuft (Fig 5**e**1) [2]. Building on this foundation, precise inhibition will suppress either the local NMDA spike or the widespread Ca^2+^ spike, thus lowering [Ca^2+^]_i_ at surrounding sites and effectively weakening or protecting synaptic weights (Fig 5**e**1-3).

A parallel line of research suggests Ca^2+^ spiking as a means of information multiplexing. This multiplexing requires decoupling of tuft and perisomatic input, the tuft determining burst-probability and the perisomatic the event (burst or spike) rate [3,4]. Balduzzi & Tononi [5] argue that an efficient information coding scheme must emphasize selective responses with bursts. An elementary experiment we ran involved training a convolutional neural network on reproducing I/O pairs of our detailed model (see [6]), and revealed bursts as highly specialized responses, explained by significantly fewer principal components.

#### References

1. Larkum ME, Zhu JJ, Sakmann B. A new cellular mechanism for coupling inputs arriving at different cortical layers. Nature [Internet]. 1999 Mar;398(6725):338–41. Available from: http://www.nature.com/articles/18686

2. Golding NL, Staff NP, Spruston N. Dendritic spikes as a mechanism for cooperative long-term potentiation. Nature [Internet]. 2002 Jul;418(6895):326–31. Available from: http://www.nature.com/articles/nature00854

3. Naud R, Sprekeler H. Sparse bursts optimize information transmission in a multiplexed neural code. Proc Natl Acad Sci [Internet]. 2018 Jul 3;115(27):E6329–38. Available from: http://www.pnas.org/lookup/doi/10.1073/pnas.1720995115

4. Payeur, A., Guerguiev, J., Zenke, F., Richards, B., & Naud R. Burst-dependent synaptic plasticity can coordinate learning in hierarchical circuits. bioRxiv. 2020;

5. Balduzzi D, Tononi G. What can neurons do for their brain? Communicate selectivity with bursts. Theory Biosci [Internet]. 2013 Mar 6;132(1):27–39. Available from: http://link.springer.com/10.1007/s12064-012-0165-0

6. Beniaguev D, Segev I, London M. Single cortical neurons as deep artificial neural networks. Neuron. 2021;109(17):2727–39.
