# Supplementary figures and images for: Burst control: Synaptic conditions for burst generation in cortical layer 5 pyramidal neurons
Source: PLoS Comput Biol. 2021 Nov 2;17(11):e1009558. doi: 10.1371/journal.pcbi.1009558 (PMC8589150; doi:10.1371/journal.pcbi.1009558)

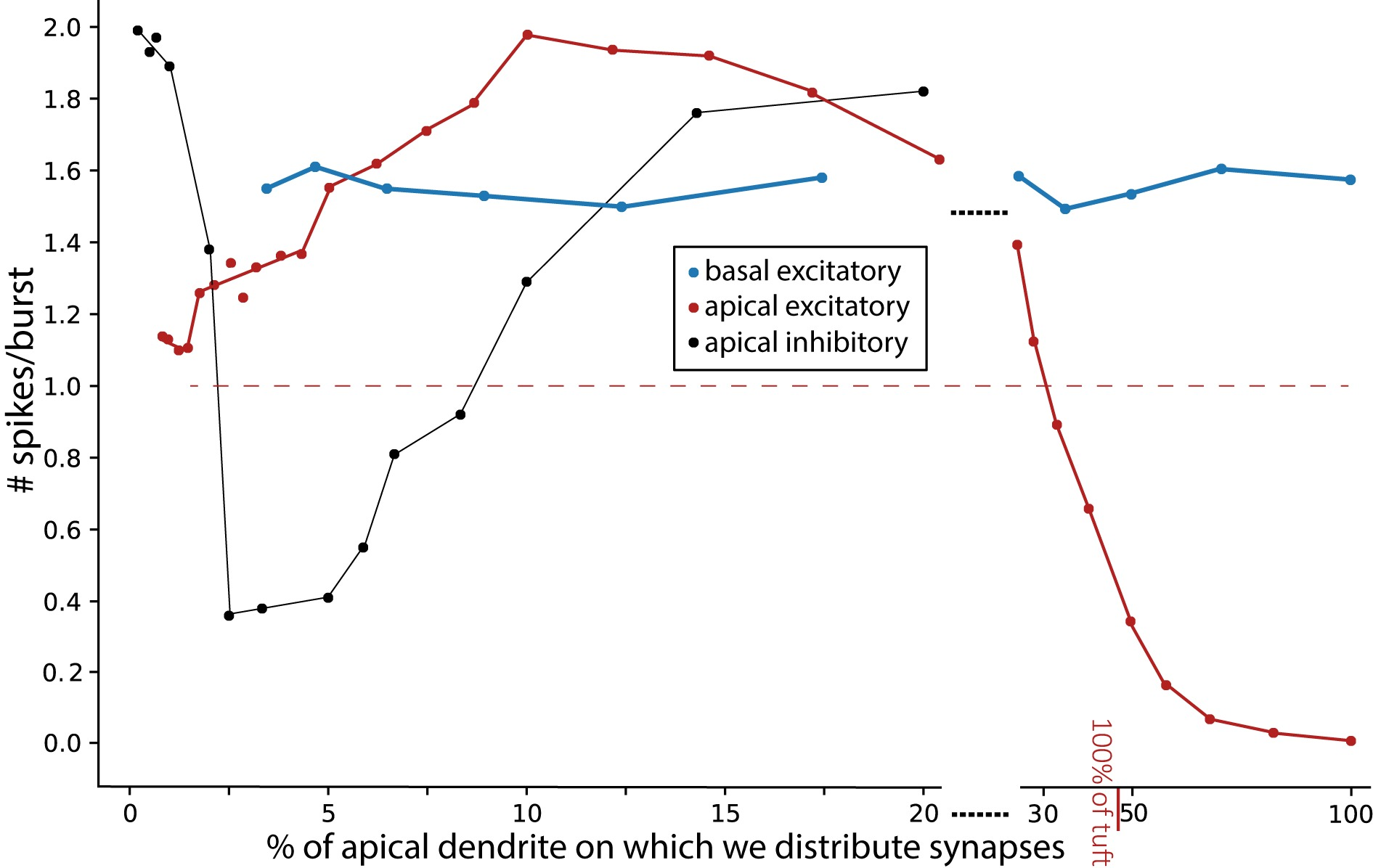

Supplement: S1 Fig — Number of spikes generated as a function of percent of dendritic tree on which excitatory alone (red apical; blue basal, apical on 5%) or inhibitory (black; apical excitation on 10%) synapses are distributed. Excitation continues over 20% at a different scale. Dashed line: apical excitation with VGCC blocked. Excitatory conditions as in Figs 1 and 2 (σ = 9 ms, Δt = 0), inhibition as in Fig 4. (TIF) [file pcbi.1009558.s004.tif]

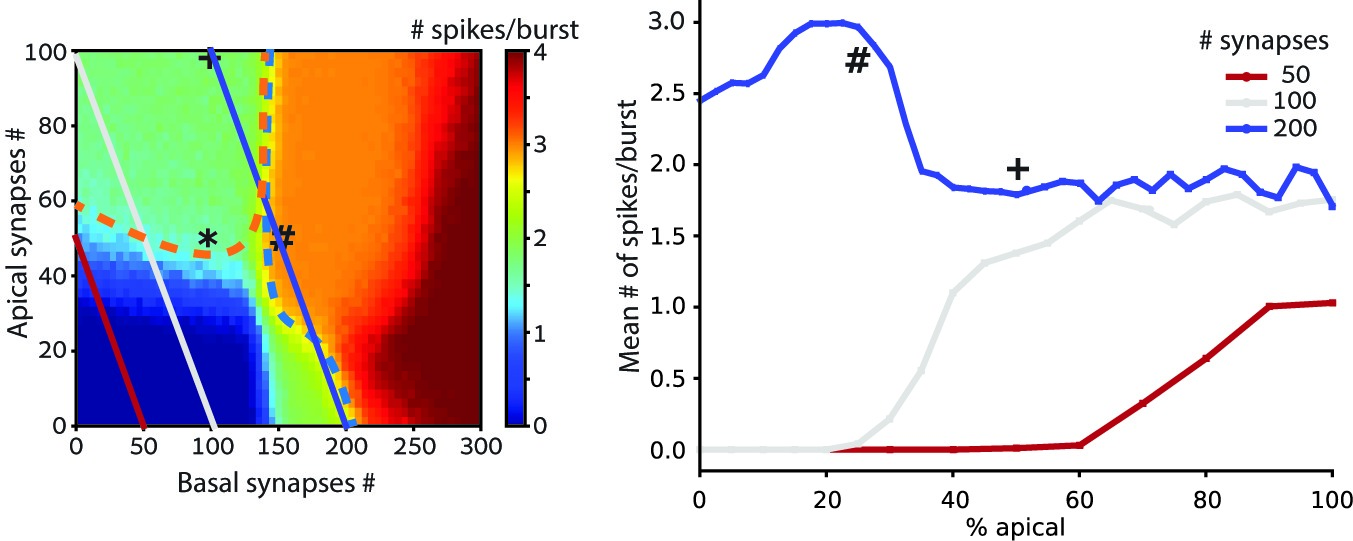

Supplement: S2 Fig — Left: heatmap of mean number of spikes per burst as a function of the number of activated synapses on the basal and apical trees, from Fig 3B. Overlaid are fixed total synapse number diagonal lines, whose profile is plotted to the right. Right: Mean number of spikes per burst, as a function of the ratio between apical and basal synapse number, for various total synapses, as plotted on the respective colored diagonals on the left. σ = 10 ms, Δt = 0. (TIF) [file pcbi.1009558.s005.tif]

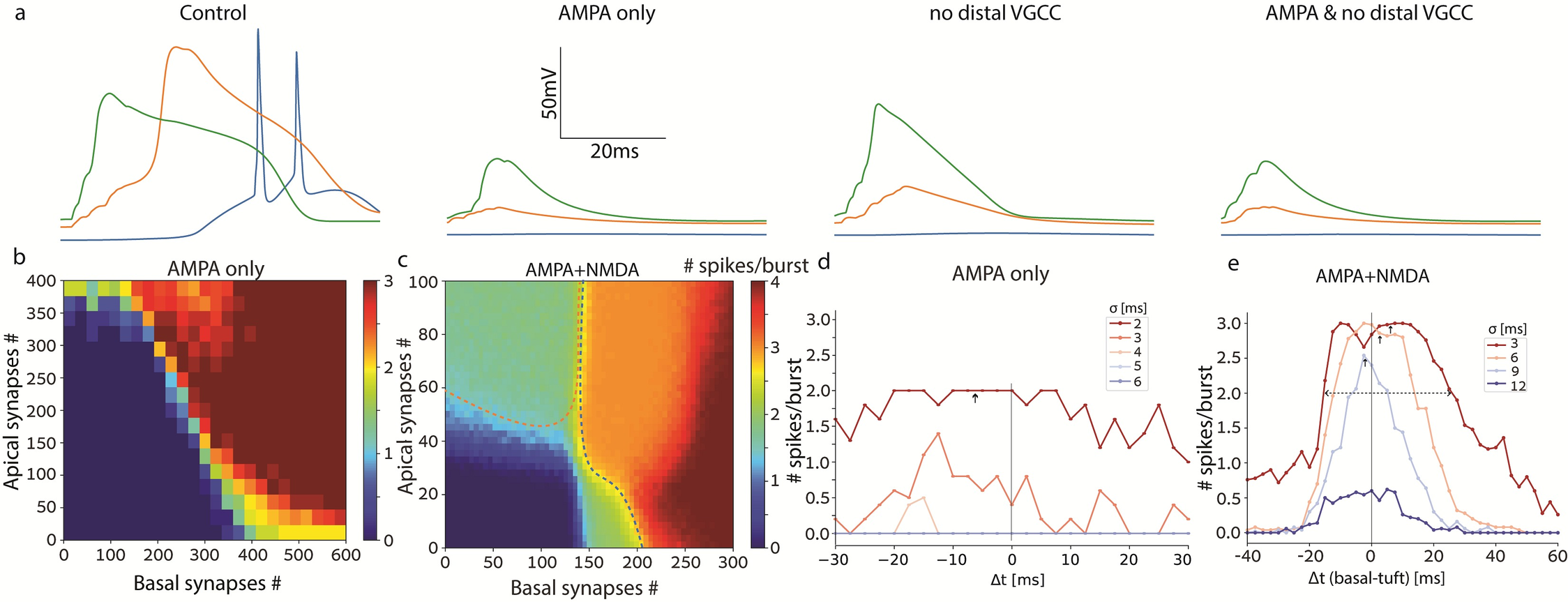

Supplement: S3 Fig — a. Somatic, nexus and distal tuft voltage traces (blue, orange and green, respectively), after activation of 30 tuft synapses (σ = 3 ms) with and without NMDA or distal VGCC. b. Heatmap of mean number of spikes per burst as a function of the number of activated synapses on the basal and apical trees, with AMPA-only synapses. The apical-only burst class vanishes with zero NMDA conductance. c. Same as b but as a control experiment with both AMPA and NMDA conductances (from Fig 2B). d. Mean number of somatic spikes per burst for a range of Δt values, without NMDA. Colored lines correspond to different σ values. e. Same as d but for control with NMDA (from Fig 2D). Note that the bursting window’s center is negative (basal input following apical input) without NMDA for small σ. (TIF) [file pcbi.1009558.s006.tif]

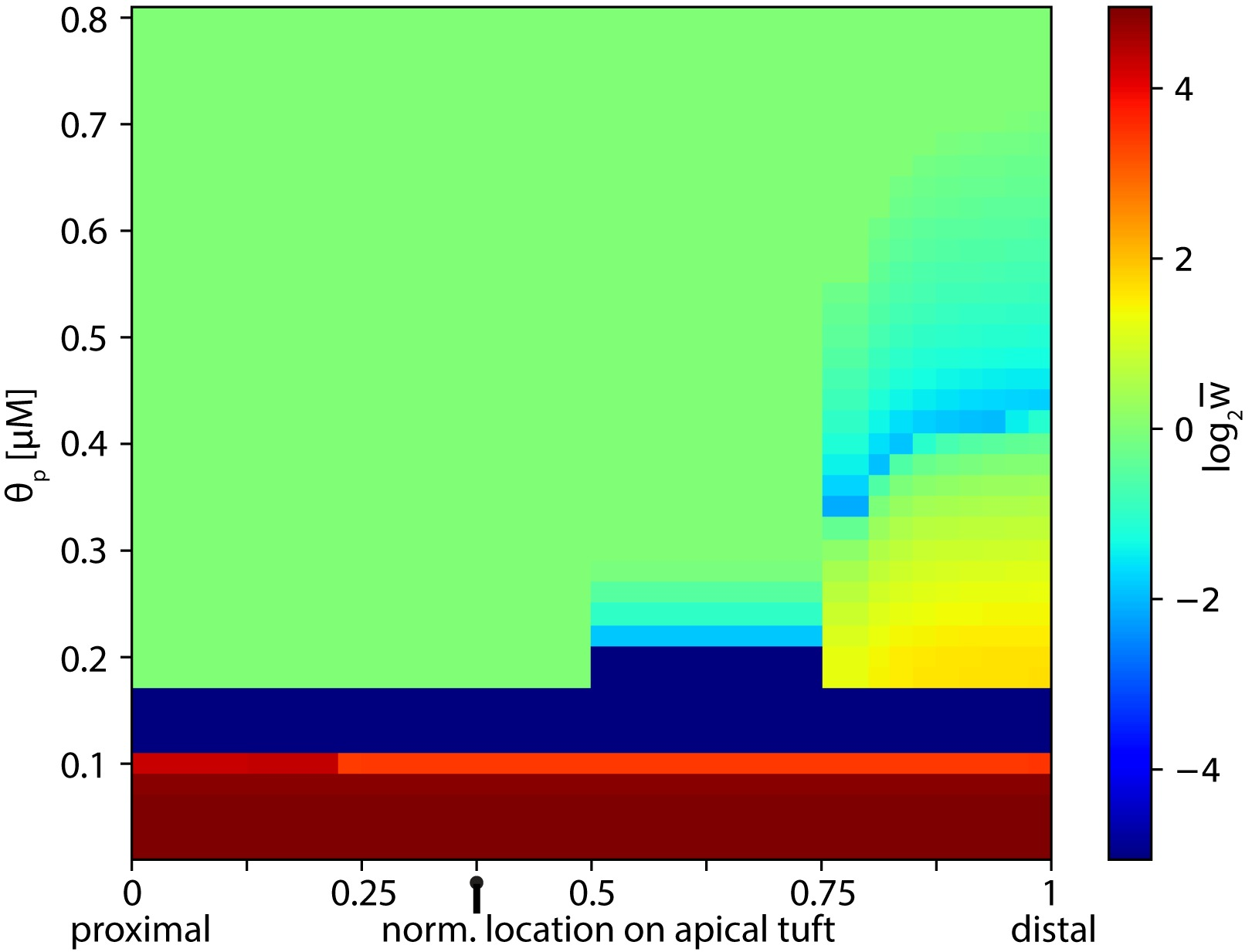

Supplement: S4 Fig — [Ca2+]i measured as in Fig 5C2 (intermediate inhibition location, Δt = 0) were used for calculating plasticity modifications as in Fig 5C2, for a range of threshold θp/d values. The heatmap represents synaptic weight after plastic modification, normalized to initial value and colored on a log2 scale (Red LTP, blue LTD, 0 –green protected). Modified excitatory synapse location is ordered on x-axis. LTP threshold θp on y-axis. LTD threshold θd is kept at a fixed ratio of 0.6 to θp. (TIF) [file pcbi.1009558.s007.tif]

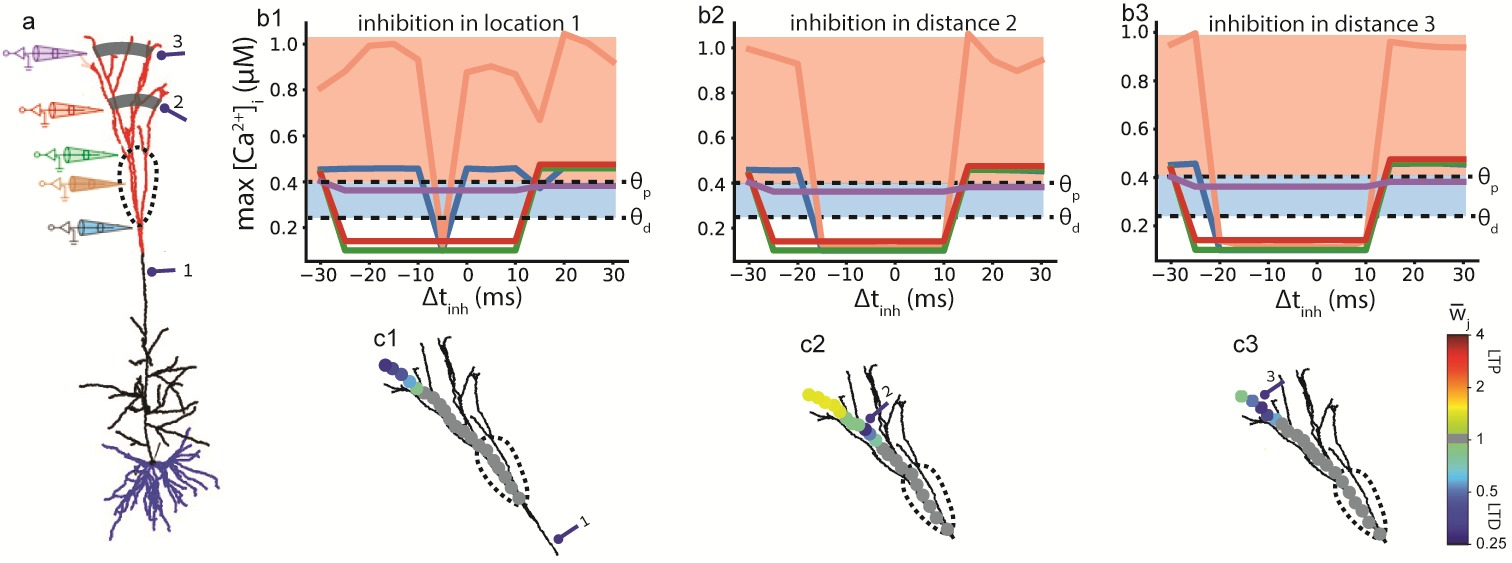

Supplement: S5 Fig — a. Model neuron as in Fig 4A. Red branches are excited. Electrodes for [Ca2+]i measurement as in Fig 5A. Inhibitory synapses at all locations of a fixed distance from the soma (a single numbered grey shaded strip). Dashed line denotes Ca2+ hotspot. b1-3. Maximal [Ca2+]i along the apical dendrite at the three locations (colored electrodes in b) as a function of Δtinh between excitation and inhibition for each inhibition location. Dashed lines mark thresholds, shadings the resulting change–LTP (red), LTD (blue). c1-3. Synaptic weights after repeated execution of the learning rule (Eq (1), Methods) for 10 input repetitions (60 seconds) and Δtinh = -5 ms at each inhibition distance # (a,b). Twenty representative excitatory tuft synapses are color plotted by their respective weights: yellow—LTP (in 2), blue—LTD, and grey—protected. (TIF) [file pcbi.1009558.s008.tif]
